# Methyltransferase-like 3/14-mediated m^6^A Silencing of GPx3 Drives Lipophagy Dysfunction and Ferroptosis Resistance in Colorectal Cancer 

**DOI:** 10.34133/research.1273

**Published:** 2026-05-11

**Authors:** Huiya Wang, Yingying Jin, Ziyi Dong, Jie Hao, Zhanhua Gao, Chong Chen, Jiayu Yang, Xinyi Wang, Fang Yang, Miao Zhang, Minghan Qiu, Hui Liu, Yaoyang Guo, Mengran Tian, Zhanyu Pan, Xipeng Zhang, Ming Gao, Yi Ba, Dongzhi Hu, Mingqing Zhang, Zhansheng Jiang, Haiyang Zhang

**Affiliations:** ^1^ Tianjin Medical University Cancer Institute and Hospital, National Clinical Research Center for Cancer, Tianjin’s Clinical Research Center for Cancer, Tianjin Key Laboratory of Digestive Cancer, Tianjin Union Medical Center, Tianjin Institute of Coloproctology, The First Affiliated Hospital of Nankai University, Tianjin, China.; ^2^ Cancer Medical Center, Peking Union Medical College Hospital, Chinese Academy of Medical Sciences, Beijing, China.; ^3^ Department of Gastroenterology, Tianjin First Central Hospital, Tianjin, China.

## Abstract

**Background:** Colorectal cancer (CRC) is a major cause of cancer morbidity and mortality worldwide, and its progression is closely associated with redox imbalance. The biological role and upstream regulation of the antioxidant enzyme glutathione peroxidase 3 (GPx3) remain poorly understood in CRC. **Methods:** This study integrated analyses from clinical CRC tissues, in vitro functional experiments, molecular biology assays, and public The Cancer Genome Atlas datasets. Techniques included gene expression profiling, *N*^6^-methyladenosine methylation analysis, mitochondrial reactive oxygen species detection, lipophagy assays, lipid droplet staining, transcriptomic and proteomic analyses, ferroptosis sensitivity testing, and in vivo xenograft and metastasis models to investigate the role and underlying mechanisms of GPx3 in CRC. In addition, lung metastatic burden was assessed using in vivo bioluminescence imaging and high-resolution micro-computed-tomography scanning. **Results:** GPx3 was substantially down-regulated in CRC tissues and cells, primarily due to *N*^6^-methyladenosine modification catalyzed by methyltransferase-like protein 3 (METTL3) and METTL14. Down-regulation of GPx3 expression elevated mitochondrial reactive oxygen species, impaired lipophagy flux, and caused excessive lipid droplet accumulation. Multiomics analyses identified Dickkopf-1 (DKK1) as a downstream target negatively regulated by GPx3 through reactive-oxygen-species-dependent β-catenin signaling. The GPx3–DKK1–solute carrier family 7 member 11 (SLC7A11) axis modulated lipid peroxidation and influenced ferroptosis sensitivity. In vivo xenograft and metastatic models showed that GPx3 suppressed tumor growth and metastasis, and these effects were partially reversed by DKK1. **Conclusion:** This study demonstrates that METTL3/14-mediated *N*^6^-methyladenosine modification is a key upstream mechanism leading to GPx3 down-regulation in CRC. Down-regulation of GPx3 expression promotes malignant progression by increasing mitochondrial oxidative stress, disrupting lipophagy, and cooperating with the DKK1–SLC7A11 pathway to reduce ferroptosis sensitivity. Targeting this regulatory axis may provide promising molecular strategies for CRC diagnosis and therapy.

## Introduction

Colorectal cancer (CRC) stands as a predominant malignancy worldwide, contributing significantly to global disease burden and fatality rates, thus constituting a critical public health issue [1,2]. Evidence from extensive epidemiological and basic research has established that obesity and its associated metabolic disorders are key risk factors for CRC [3,4]. At the molecular level, the pathophysiological progression of CRC is intricately linked with significant cellular metabolic reprogramming, a core feature of which is the aberrant accumulation of intracellular lipid droplets (LDs) [5,6]. These perturbations in lipid homeostasis are intricately linked to the malignant progression of CRC, encompassing phenotypes such as invasiveness, metastatic spread, and therapeutic resistance [7]. Consequently, a thorough elucidation of the regulatory networks governing lipid metabolic reprogramming in CRC is of profound theoretical and clinical importance for understanding tumor biology and developing novel therapeutic strategies.

Lipophagy, as a form of selective autophagy, is a central mechanism by which cells degrade LDs via the lysosomal pathway to maintain intracellular lipid homeostasis. The dysregulation of this process is a critical factor leading to the excessive accumulation of LDs within tumor cells [8,9]. As the primary source of intracellular reactive oxygen species (ROS), mitochondria generate mitochondrial ROS (mtROS), which function as key upstream signaling molecules in the regulation of lipophagic activity. An imbalance in mtROS levels can significantly inhibit lipophagic flux, thereby exacerbating LD accumulation. Accumulation of peroxidation-prone free lipids can provide substrates for lipid peroxidation and sensitize cells to ferroptosis, while, under certain contexts, also contributing to the maintenance of malignant tumor phenotypes [10–15]. Therefore, dissecting the molecular networks that regulate the mtROS–lipophagy axis is a crucial entry point for revealing the mechanisms of metabolic adaptation in CRC.

The remodeling of redox homeostasis is a hallmark of malignancy [16,17]. ROS exhibit a dual function in tumor development: Moderate increases in ROS serve as signaling mediators that facilitate cancer cell proliferation and metastatic spread. This is achieved through the induction of DNA damage and the subsequent up-regulation of protumorigenic gene expression. Conversely, exceedingly high levels of ROS can induce oxidative-stress-induced cell death, thereby exerting an anticancer effect. Accordingly, a sophisticated intracellular antioxidant system is essential for maintaining ROS balance [18]. Glutathione (GSH) peroxidase 3 (GPx3), a key member of the GSH peroxidase family, possesses potent antioxidant activity [19]. The expression of GPx3 is significantly down-regulated in various malignancies, including lung, breast, and esophageal cancers. In these cancers, it functions to inhibit cancer cell proliferation and invasion [20,21]. Given that the unique physiological function of the intestine subjects it to chronic high-oxidative-stress environments, maintaining redox homeostasis is of paramount importance. However, the specific expression patterns, prognostic value, and mechanisms of action of GPx3 in the context of CRC remain to be systematically investigated.

The precise downstream molecular network through which GPx3 exerts its tumor-suppressive function remains largely unclear. Our attention has been drawn to Dickkopf-1 (DKK1), a classical inhibitor of the Wnt signaling pathway, which is paradoxically overexpressed in CRC and plays a complex protumorigenic role [22–25]. It has been reported that DKK1 can up-regulate the expression of solute carrier family 7 member 11 (SLC7A11), a core component of the cystine/glutamate antiporter that represents a rate-limiting step in cellular GSH synthesis and subsequent resistance to ferroptosis [26,27]. As an antioxidant enzyme, GPx3 directly influences the cellular oxidative stress state [28,29]. Thus, a critical scientific question that needs to be elucidated is whether a regulatory link exists between the antioxidant function of GPx3 and the DKK1–SLC7A11 signaling axis and how such a connection ultimately impacts the ferroptotic fate of CRC cells. Concurrently, the upstream regulatory mechanisms leading to the down-regulation of GPx3 in CRC also represent a key unresolved issue. *N*^6^-methyladenosine (m^6^A), the most abundant and reversible chemical mark on mRNA, is mainly deposited by the methyltransferase-like protein 3/14 (METTL3/14) methyltransferase complex and exerts broad regulatory effects on gene expression by modulating mRNA stability, alternative splicing, and translational output [29–32]. Our bioinformatic analysis predicts potential m^6^A modification sites on the GPx3 transcript, offering a new direction for investigating its mechanism of inactivation.

This work investigates how GPx3 is expressed in CRC and explores its underlying mechanisms. Specifically, we will investigate the regulatory effects of GPx3 on both total cellular and mtROS levels. We will further assess its function in modulating lipophagic flux, influencing LD deposition, and determining the sensitivity of tumor cells to ferroptosis. Concurrently, we will conduct an in-depth analysis of the underlying molecular mechanisms, with the goal of providing new insights and identifying novel therapeutic targets for the diagnosis and treatment of CRC.

## Results

### GPx3 expression is significantly reduced in CRC

**Fig. 1.**
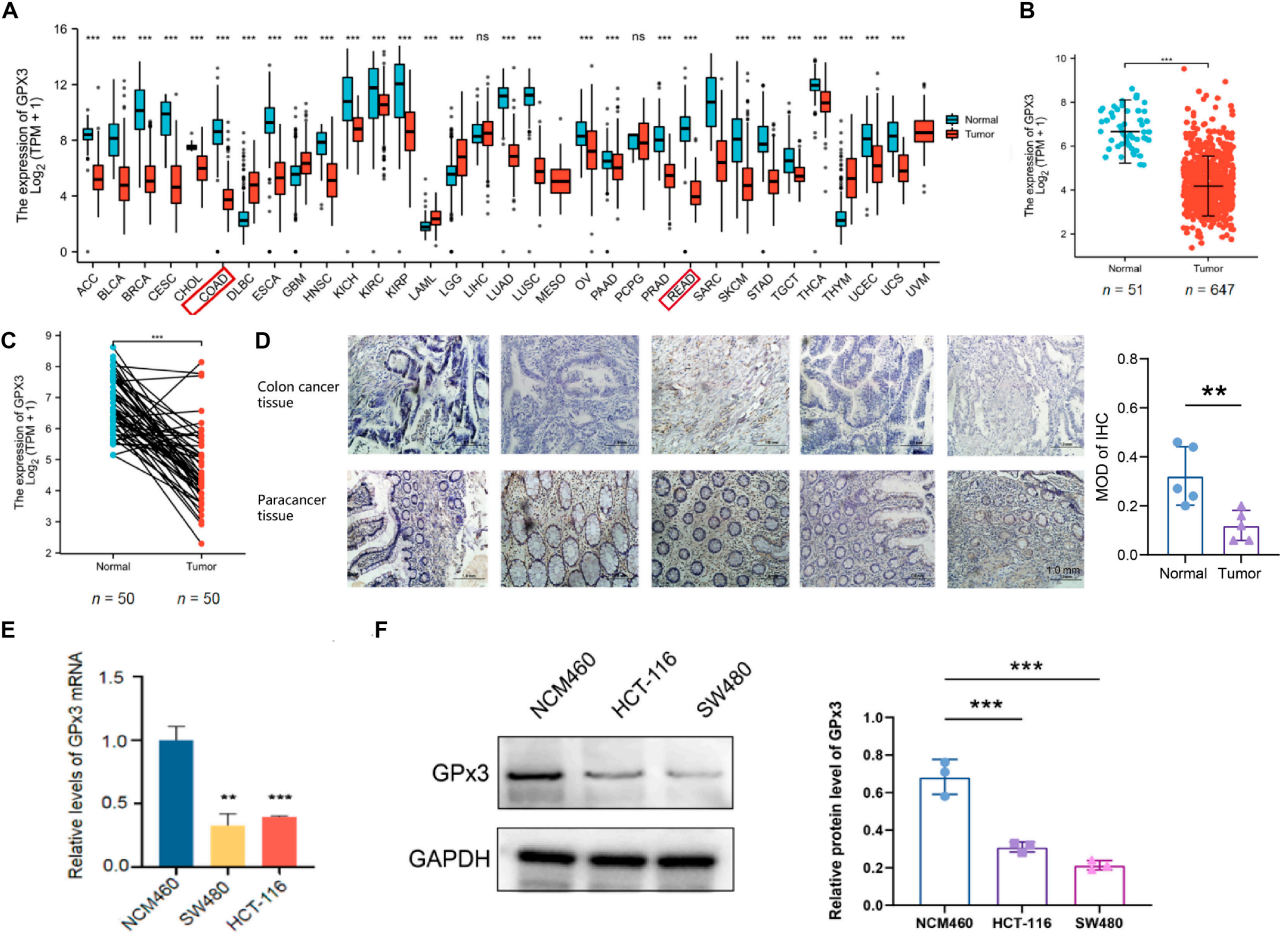
Glutathione peroxidase 3 (GPx3) is down-regulated in colorectal cancer (CRC). (A) Pan-cancer analysis of GPx3 expression using The Cancer Genome Atlas (TCGA) datasets. ACC, adrenocortical carcinoma; BLCA, bladder urothelial carcinoma; BRCA, breast invasive carcinoma; CESC, cervical squamous cell carcinoma and endocervical adenocarcinoma; CHOL, cholangiocarcinoma; COAD, colon adenocarcinoma; DLBC, lymphoid neoplasm diffuse large B cell lymphoma; ESCA, esophageal carcinoma; GBM, glioblastoma multiforme; HNSC, head and neck squamous cell carcinoma; KICH, kidney chromophobe; KIRC, kidney renal clear cell carcinoma; KIRP, kidney renal papillary cell carcinoma; LAML, acute myeloid leukemia; LGG, brain lower grade glioma; LIHC, liver hepatocellular carcinoma; LUAD, lung adenocarcinoma; LUSC, lung squamous cell carcinoma; MESO, mesothelioma; OV, ovarian serous cystadenocarcinoma; PAAD, pancreatic adenocarcinoma; PCPG, pheochromocytoma and paraganglioma; PRAD, prostate adenocarcinoma; READ, rectum adenocarcinoma; SARC, sarcoma; SKCM, skin cutaneous melanoma; STAD, stomach adenocarcinoma; TGCT, testicular germ cell tumors; THCA, thyroid carcinoma; THYM, thymoma; UCEC, uterine corpus endometrial carcinoma; UCS, uterine carcinosarcoma; UVM, uveal melanoma. (B) GPx3 levels were significantly reduced in CRC tumor samples (*n* = 647) compared with adjacent normal tissues (n = 51) in the unpaired TCGA cohort. (C) GPx3 expression is significantly lower in CRC tumor tissues compared to their matched normal counterparts in paired samples (*n* = 50) from the TCGA dataset. (D) Representative immunohistochemical (IHC) images of GPx3 in CRC tissues and adjacent normal mucosa and quantification of mean optical density (MOD). (E) GPx3 mRNA levels in NCM460 and CRC cell lines (SW480 and HCT-116) assessed byquantitative reverse transcription polymerase chain reaction (qRT-PCR). (F) GPx3 protein expression in these cell lines analyzed by Western blot. Data are presented as means ± SD. Statistical significance between 2 groups was determined by *t* test, and comparisons among multiple groups were performed using ANOVA analysis; **P < 0.01 and ***P < 0.001. ns, not significant.

To investigate GPx3 expression patterns across human cancers, we examined The Cancer Genome Atlas (TCGA) Program RNA sequencing profiles. The results showed that GPx3 expression was frequently down-regulated in multiple tumor types, including CRC (Fig. 1A). Analysis of TCGA CRC datasets showed markedly reduced GPx3 expression in tumor tissues relative to adjacent normal tissues in both unpaired (Fig. 1B) and paired (Fig. 1C) comparisons. To validate these observations, we performed immunohistochemical (IHC) staining on clinical specimens obtained from surgical resections at our institution. IHC staining further verified that GPx3 immunoreactivity was noticeably diminished in CRC tissues compared with their matched adjacent normal counterparts (Fig. 1D). Kaplan–Meier survival analysis revealed that patients with high GPx3 expression had significantly improved overall survival compared with those with low GPX3 expression (hazard ratio = 0.42, 95% confidence interval: 0.18 to 0.98, *P* = 0.046; Fig. S1). In line with the tissue findings, quantitative reverse transcription polymerase chain reaction (qRT-PCR) revealed substantially reduced GPx3 transcript levels in CRC cell lines compared with the nonmalignant colonic epithelial cell line NCM460 (Fig. 1E). Furthermore, Western blot analysis revealed a corresponding decrease in GPx3 protein expression in both CRC cell lines (Fig. 1F). Taken together, these findings indicate that GPx3 is consistently reduced in CRC at both the mRNA and protein levels.

### Effects of GPx3 on proliferation and metastatic capacity of CRC

**Fig. 2.**
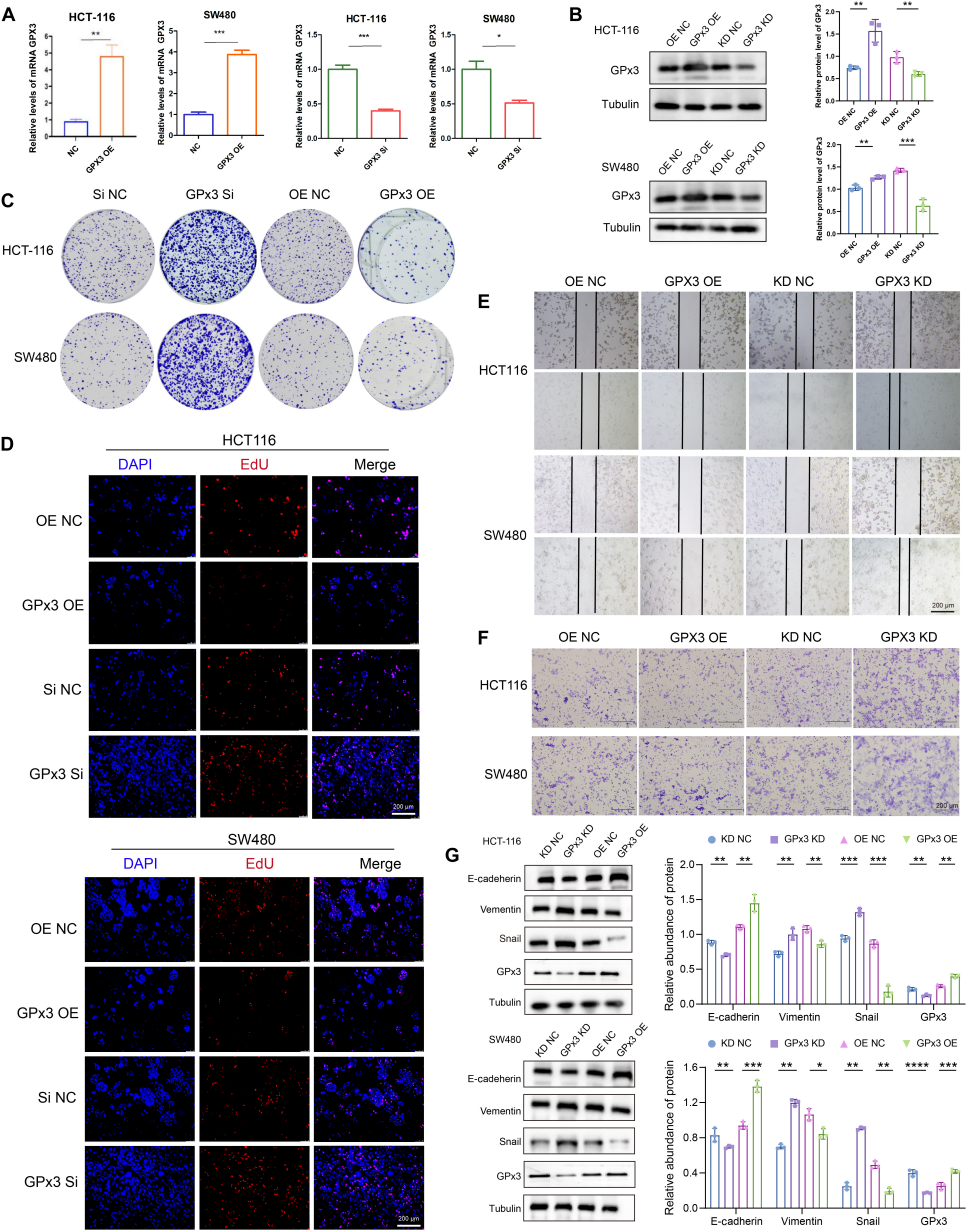
Glutathione peroxidase 3 (GPx3) restrains colorectal cancer (CRC) cell growth and metastasis. (A and B) The efficiency of GPx3 overexpression (GPx3 OE) and siRNA-mediated knockdown (Si) in HCT-116 and SW480 cells was confirmed by quantitative reverse transcription polymerase chain reaction (qRT-PCR) and Western blot. (C) Colony formation assays showing the effect of GPx3 on the clonogenic potential of CRC cells. (D) 5-Ethynyl-2′-deoxyuridine (EdU) incorporation assays assessing the impact of GPx3 on CRC cell proliferation. (E) Wound-healing assays demonstrating the effect of GPx3 on cell migration. (F) Transwell assays showing the effect of GPx3 on cell migration. (G) Western blot analysis of epithelial–mesenchymal transition (EMT) markers (E-cadherin, Vimentin, and Snail) following GPx3 modulation. Data are presented as means ± SD; *P < 0.01, **P < 0.01, ***P < 0.001, and ****P < 0.0001 in unpaired *t* test.

To explore the functional role of GPx3 in CRC, we transiently transfected SW480 and HCT-116 cells with a GPx3 overexpression plasmid (GPx3 OE) or GPx3-targeting small interfering RNA (siRNA) for knockdown of GPx3 (GPx3 KD), together with their respective controls. Transfection efficiency was verified by qRT-PCR (Fig. 2A) and Western blot (Fig. 2B). We next performed a series of functional assays to evaluate the effects of GPx3 on malignant behaviors. The clonogenic formation assay showed that GPx3 OE significantly inhibited colony formation in both cell lines, while GPx3 KD increased it (Fig. 2C). These results were supported by 5-ethynyl-2′-deoxyuridine (EdU) incorporation assays, which revealed a lower percentage of EdU-positive cells upon GPx3 OE and a higher percentage upon GPx3 silencing (Fig. 2D), confirming that GPx3 suppresses CRC cell proliferation.

We further assessed the impact of GPx3 on migration using wound-healing and Transwell assays. Overexpression of GPx3 (GPx3 OE) delayed wound closure, whereas GPx3 KD accelerated it (Fig. 2E). Similarly, Transwell migration assays showed reduced cell migration in GPx3 OE cells and enhanced migration following GPx3 KD (Fig. 2F). To investigate the mechanism by which GPx3 inhibits migration, we analyzed key epithelial–mesenchymal transition (EMT) markers. Western blotting showed that GPx3 OE elevated E-cadherin levels while reducing Snail and Vimentin expression. Conversely, GPx3 KD produced the opposite effect (Fig. 2G). These findings suggest that GPx3 attenuates the migratory capacity of CRC cells by suppressing EMT.

### GPx3 reduces mtROS and LD accumulation through regulation of lipophagy in CRC

**Fig. 3.**
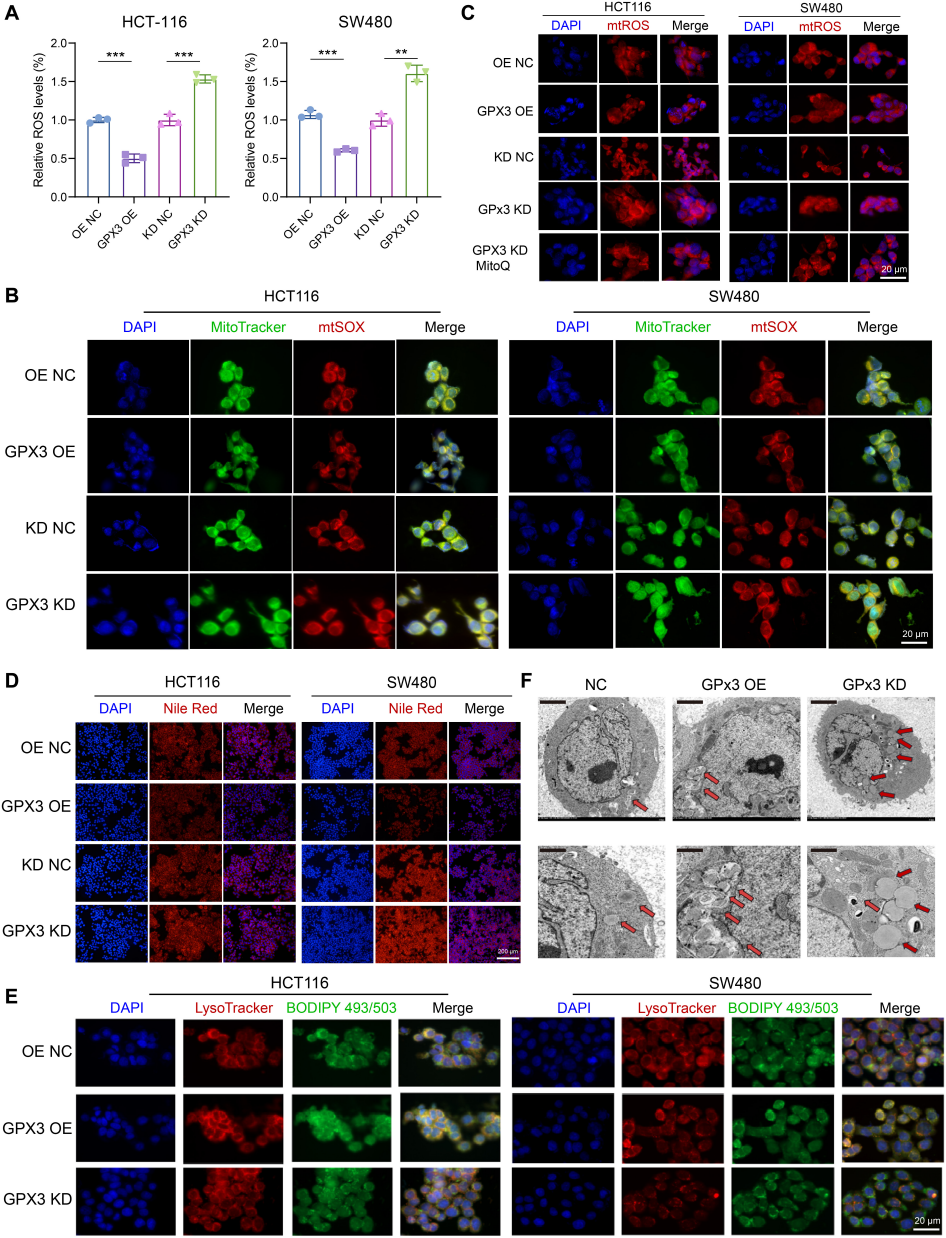
Glutathione peroxidase 3 (GPx3) reduces mitochondrial reactive oxygen species (mtROS) and lipid droplet (LD) accumulation by regulating lipophagy. (A) ROS-DF probing analysis of total ROS levels in HCT-116 and SW480 cells with different GPx3 expression levels. (B) Immunofluorescence colocalization analysis of mtROS (mtSOX, red) and mitochondria (MitoTracker, green). (C) Immunofluorescence showing mtROS levels in GPx3-knockdown cells with or without treatment with the mitochondria-targeted antioxidant MitoQ. (D) Nile Red staining to visualize intracellular LDs. (E) Immunofluorescence colocalization analysis of lysosomes (LysoTracker, red) and LDs (BODIPY 493/503, green) to assess lipophagic flux. (F) Transmission electron microscopy (TEM) images showing LDs (red arrows) and autophagic structures in colorectal cancer (CRC) cells with different GPx3 expression levels. Scale bars, 2 μm (top) and 1 μm (bottom). Data are presented as means ± SD; **P < 0.01 and ***P < 0.001 in unpaired *t* test.

GPx3, a key intracellular antioxidant that preserves redox homeostasis by scavenging excess ROS [33–35], demonstrably lowers ROS in CRC cells, as ROS detection fluorescence (ROS-DF) probing revealed significantly reduced fluorescence upon GPx3 OE and, conversely, heightened signals after GPx3 KD, thereby underscoring its central role in oxidative stress regulation (Fig. 3A). Mitochondria are the primary cellular sources of ROS, with mtROS playing a critical role in modulating cellular oxidative stress [36–38]. To investigate how GPx3 influences mtROS levels, we performed immunofluorescence colocalization assays using mtSOX, a mitochondria-specific ROS probe that emits red fluorescence, together with MitoTracker, a mitochondria-targeted indicator that emits green fluorescence. Notably, GPx3 OE cells exhibited minimal mtSOX–MitoTracker overlap, whereas GPx3 KD intensified the yellow colocalization signal, indicating effective suppression of mtROS by GPx3 (Fig. 3B). Furthermore, GPx3 up-regulation attenuates mtSOX fluorescence intensity, indicating a suppression of mtROS levels, while down-regulation amplifies it. In addition, importantly, treatment with MitoQ, a mitochondria-targeted compound specifically designed to mitigate mtROS, successfully reversed the elevated mtROS levels induced by GPx3 deficiency (Fig. 3C).

Emerging evidence has demonstrated that mtROS modulate lipid metabolism and autophagy in tumor cells, and changes in mtROS profoundly affect LD autophagy (lipophagy), consequently altering lipid deposition in cancer cells [39–41]. Increased LD accumulation has been observed in tumor cells, which impairs lipid metabolism and promotes malignant progression in colon cancer cells [42–44]. To evaluate the impact of GPx3 on lipid accumulation in colon cancer cells. Nile Red staining demonstrated a significant reduction of LDs upon GPx3 OE, in contrast to an elevation following GPx3 KD (Fig. 3D). Lipophagy, the autophagy-mediated lysosomal breakdown of LDs into free fatty acids utilized by cells, represents an essential metabolic mechanism regulating LDs in tumor cells [45]. To explore GPx3’s role in modulating lipophagy in colon cancer cells, we performed immunofluorescence colocalization assays utilizing LysoTracker (lysosome marker) and BODIPY 493/503 (LD marker). The result revealed that GPx3 enhances lipophagic flux, as evidenced by increased lysosome–lipid droplet colocalization after GPx3 OE and the opposite pattern following its knockdown (Fig. 3E). Transmission electron microscopy (TEM) corroborated these findings, showing fewer LDs and more autophagosome-encapsulated droplets in GPx3-overexpressing cells, whereas GPx3 KD cells accumulated abundant droplets with scant autophagic structures, emphasizing GPx3’s role in reducing lipid accumulation through enhanced lipophagy (Fig. 3F).

To further distinguish impaired lipophagy from enhanced lipid synthesis, we performed autophagy flux assays using the lysosomal inhibitor bafilomycin A1 (Fig. S2). In parallel, LD staining revealed increased LD retention following bafilomycin A1 treatment, further supporting impaired lipophagic degradation rather than enhanced lipid synthesis (Fig. S3). Collectively, these results indicate that GPx3 mitigates mtROS, which impedes the binding of autophagosomes and lysosomes, thereby promoting lipophagy-dependent LD degradation, reducing lipid accumulation, and potentially restraining CRC progression.

### DKK1 is a downstream target negatively regulated by GPx3 in CRC

**Fig. 4.**
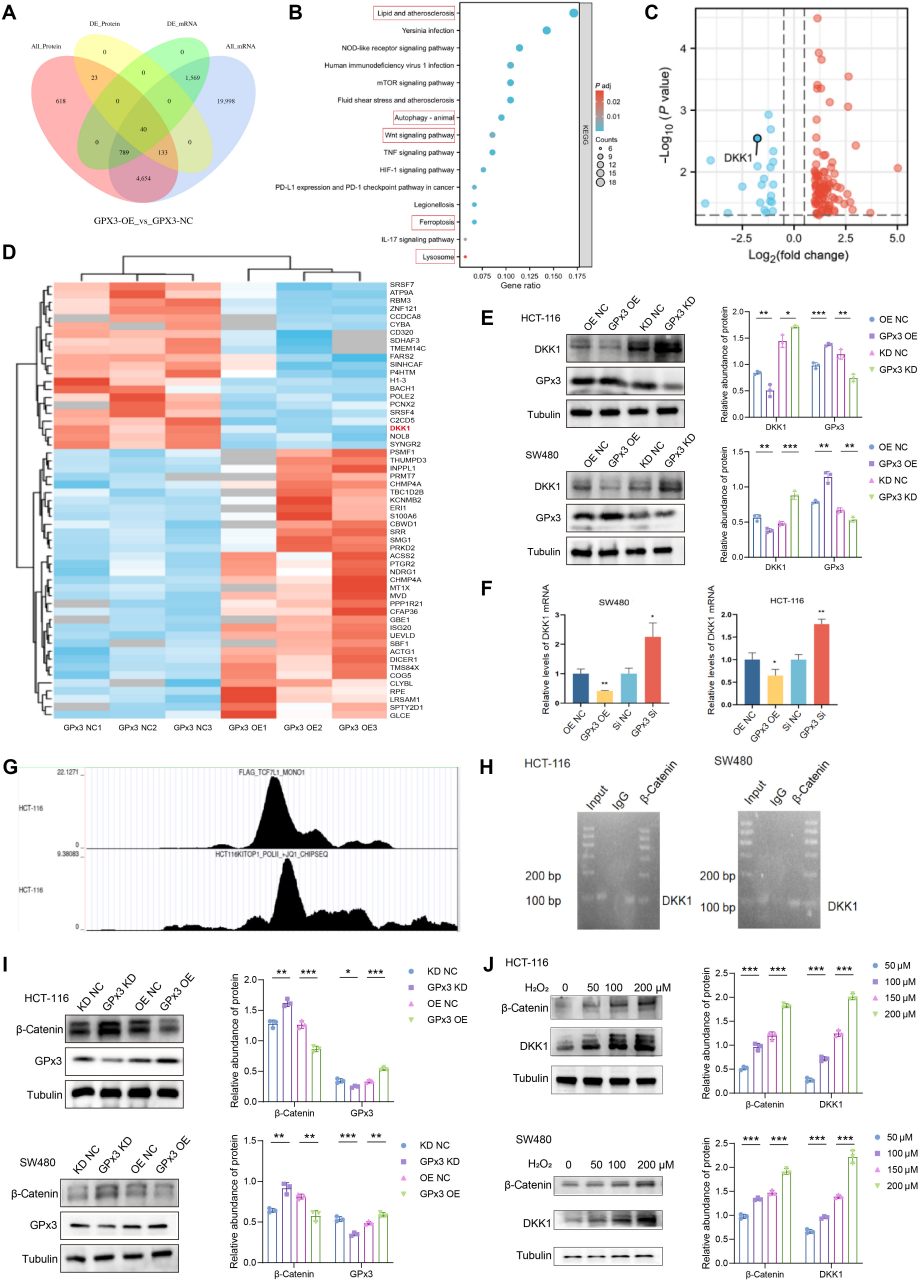
Glutathione peroxidase 3 (GPx3) negatively regulates Dickkopf-1 (DKK1) via reactive oxygen species (ROS)-mediated β-catenin signaling. (A) Venn diagram of differentially expressed genes/proteins from transcriptomic and proteomic analyses. (B) Kyoto Encyclopedia of Genes and Genomes (KEGG) pathway enrichment analysis of differentially expressed genes. NOD, nucleotide-binding oligomerization domain; mTOR, mechanistic target of rapamycin; TNF, tumor necrosis factor; HIF-1, hypoxia-inducible factor 1; PD-L1, programmed cell death ligand 1; PD-1, programmed cell death protein 1; IL-17, interleukin 17. (C and D) Volcano plot and heatmap showing significant down-regulation of DKK1 in GPx3-overexpressing cells. (E and F) Validation of the negative regulation of DKK1 by GPx3 using Western blot and quantitative reverse transcription polymerase chain reaction (qRT-PCR). (G) The Cistrome database indicates a β-catenin binding peak in the DKK1 promoter region. (H) Chromatin immunoprecipitation (ChIP)–quantitative polymerase chain reaction (qPCR) reveals β-catenin binding to the DKK1 promoter. (I) Western blot showing the negative regulation of β-catenin protein expression by GPx3. (J) Western blot showing a dose-dependent increase in β-catenin and DKK1 protein levels after H_2_O_2_ treatment. Data are presented as means ± SD; *P < 0.05, **P < 0.01 and ***P < 0.001 in unpaired *t* test.

To investigate the mechanism by which GPx3 suppresses CRC progression, we performed integrated transcriptomic and proteomic profiling of HCT-116 cells GPx3 OE versus vector controls. This analysis identified a set of differentially expressed genes and proteins (Fig. 4A). Kyoto Encyclopedia of Genes and Genomes (KEGG) pathway enrichment revealed that these changes were associated with lipid metabolism, autophagy, Wnt signaling, ferroptosis, and lysosomal pathways (Fig. 4B). Among the significantly altered genes, DKK1—a known Wnt pathway inhibitor linked to poor prognosis in multiple cancers—was notably down-regulated upon GPx3 OE, as visualized in volcano plot and heatmap analyses (Fig. 4C and D). Given that elevated DKK1 has been shown to promote tumor progression by up-regulating SLC7A11 and suppressing ferroptosis, we further validated its regulation by GPx3.

Western blot analysis confirmed that GPx3 OE reduced, while GPx3 KD increased, DKK1 protein levels in HCT-116 and SW480 cells (Fig. 4E). Consistent with this, qRT-PCR demonstrated corresponding changes in DKK1 mRNA expression (Fig. 4F), indicating transcriptional regulation. Since GPx3 modulates intracellular ROS (Fig. 3A) and ROS influences gene transcription, we hypothesized that GPx3 may regulate DKK1 through redox-sensitive transcriptional mechanisms. Previous studies indicate that β-catenin transcriptionally activates DKK1 [46–49]. Using the Cistrome Data Browser, we identified potential β-catenin binding sites in the DKK1 promoter (Fig. 4G), which was confirmed by chromatin immunoprecipitation (ChIP)–qPCR showing significant promoter enrichment with β-catenin antibody compared to immunoglobulin G (IgG) control (Fig. 4H).

We next examined whether GPx3 influences β-catenin expression. Western blot revealed that GPx3 OE decreased, while GPx3 KD increased, β-catenin protein levels in both CRC cell lines (Fig. 4I). We therefore proposed that GPx3 down-regulates DKK1 by reducing ROS and subsequently decreasing β-catenin. To test this, we treated cells with H_2_O_2_ to elevate ROS. Results showed a dose-dependent increase in both β-catenin and DKK1 protein levels (Fig. 4J), supporting the role of ROS in activating this axis. In summary, multiomics profiling combined with functional validation establishes that GPx3 transcriptionally represses DKK1 by modulating intracellular ROS levels and down-regulating β-catenin expression.

### DKK1 is up-regulated in CRC and promotes malignant phenotypes

**Fig. 5.**
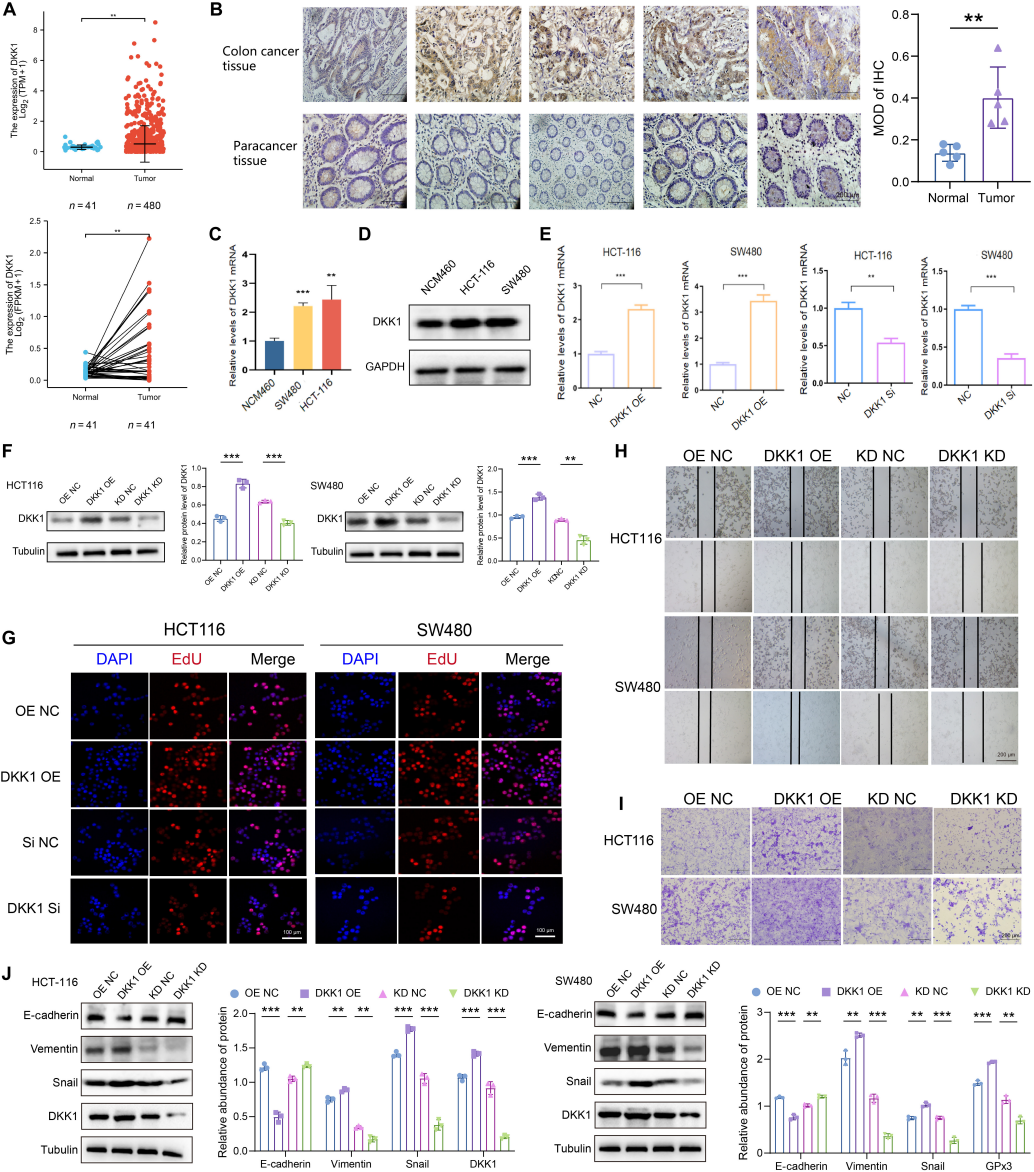
Dickkopf-1 (DKK1) is elevated in colorectal cancer (CRC) and enhances cell proliferation and metastasis. (A) DKK1 expression is significantly elevated in paired CRC tumor versus adjacent normal tissues (*n* = 41, The Cancer Genome Atlas [TCGA], fragments per kilobase of transcript per million mapped reads [FPKM]). (B) Representative immunohistochemical (IHC) images showing DKK1 staining in CRC and adjacent normal tissues. (C and D) DKK1 mRNA and protein levels in NCM460, HCT-116, and SW480 cells assessed by quantitative reverse transcription polymerase chain reaction (qRT-PCR) and Western blot. (E and F) The efficiency of DKK1 overexpression (DKK1 OE) and knockdown (Si) was validated by qRT-PCR and Western blot. (G) 5-Ethynyl-2′-deoxyuridine (EdU) assays showing the effect of DKK1 on CRC cell proliferation. (H and I) Wound-healing and Transwell assays demonstrating the effect of DKK1 on cell migration. (J) Western blot analysis of epithelial–mesenchymal transition (EMT) markers following DKK1 modulation. Data are presented as means ± SD; **P < 0.01 and ***P < 0.001 in unpaired *t* test.

To assess the clinical and functional significance of DKK1 in CRC, we first examined its expression using public and experimental data. Analysis of TCGA datasets indicated that DKK1 mRNA expression was markedly elevated in CRC tissues compared with normal mucosa (Fig. 5A). IHC of clinical samples confirmed stronger DKK1 protein expression in tumors compared with matched adjacent nontumor tissues (Fig. 5B). In agreement with these observations, DKK1 mRNA and protein levels were both higher in CRC cell lines than in the nonmalignant colonic epithelial cell line NCM460 (Fig. 5C and D).

We next explored the functional consequences of altering DKK1 expression. Transient transfection with a DKK1 overexpression plasmid (DKK1 OE) or DKK1-targeting siRNA (DKK1 KD) was performed in CRC cells, with qRT-PCR and Western blot assays confirming transfection efficiency (Fig. 5E and F). EdU incorporation assays indicated that DKK1 OE enhanced cell proliferation, while DKK1 KD suppressed it (Fig. 5G). In wound-healing assays, DKK1 OE accelerated scratch closure, and its silencing impeded cell migration (Fig. 5H). These results were supported by Transwell migration assays, which showed increased cell migration upon DKK1 OE and decreased migration following DKK1 KD (Fig. 5I). To further explore how DKK1 enhances migratory activity, we examined key EMT markers [50,51]. Western blot analysis revealed that DKK1 OE down-regulated E-cadherin and up-regulated Snail and Vimentin. Conversely, DKK1 KD elevated E-cadherin levels while decreasing Snail and Vimentin expression (Fig. 5J). These data imply that DKK1 enhances the migratory capacity of CRC cells by inducing EMT. Taken together, these results establish that DKK1 is frequently up-regulated in CRC and contributes to tumor progression by promoting proliferation and migration, likely through facilitation of EMT.

### GPx3–DKK1–SLC7A11 axis affects ferroptosis sensitivity in CRC

**Fig. 6.**
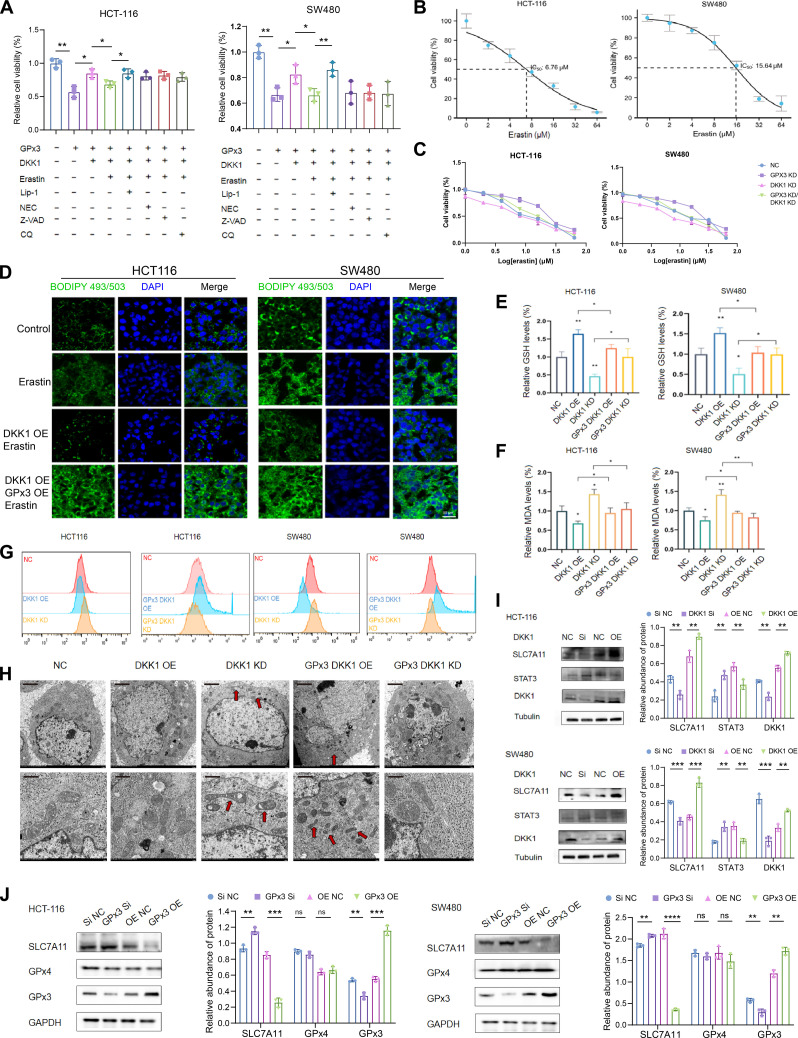
The glutathione peroxidase 3 (GPx3)–Dickkopf-1 (DKK1)–SLC7A11 axis affects ferroptosis sensitivity in colorectal cancer (CRC). (A) The effects of altering the expression of GPx3 and DKK1 in colon cancer cells, combined with apoptosis inhibitors (Z-VAD-FMK, 1 μM), necroptosis inhibitors (NEC, 1 μM), ferroptosis inhibitors (Lip-1, 1 μM), autophagy inhibitors (CQ, 10 μM), and ferroptosis inducers (Erastin, 5 μM) on the viability of colon cancer cells were investigated. (B) Half-maximal inhibitory concentration (IC_50_) values of Erastin in HCT-116 and SW480 cells determined by Cell Counting Kit-8 (CCK-8) assay. (C) Cell viability curves showing the sensitivity of different cells to Erastin. (D) Fluorescence microscopy of fatty acid uptake (BODIPY 493/503 staining) in cells treated with Erastin. (E and F) Detection of measurement of glutathione (GSH) and malondialdehyde (MDA) levels indicating the influence of GPx3 and DKK1 on ferroptosis sensitivity in CRC cells. (G) Flow cytometry analysis of intracellular lipid reactive oxygen species (ROS) levels. (H) Transmission electron microscopy (TEM) analysis illustrating alterations in mitochondrial morphology of colonic cells upon modulation of GPx3 and DKK1. Scale bars, 2 μm (top) and 1 μm (bottom). (I) Western blot analysis of SLC7A11, STAT3, and DKK1 protein expression. (J) Western blot analysis of SLC7A11 and GPx4 following GPx3 modulation. Data are presented as means ± SD. Statistical significance between 2 groups was determined by *t* test, and comparisons among multiple groups were performed using ANOVA analysis; *P < 0.05, **P < 0.01, and ***P < 0.001.

To validate the impact of the GPx3–DKK1 axis on ferroptosis sensitivity, we tested the sensitivity of HCT-116 and SW480 cells to Erastin via Cell Counting Kit-8 (CCK-8) assays (Fig. 6A and B). The data showed that, when compared with the control group, DKK1 KD significantly increased the sensitivity of both cell lines to Erastin. However, when both GPx3 and DKK1 were knocked down simultaneously, the lethal effect of Erastin on the tumor cells was significantly diminished (Fig. 6C). To further address the source of lipid accumulation, we performed BODIPY 493/503 staining to assess neutral lipid content and fatty acid uptake. Fluorescence imaging showed that Erastin treatment increased lipid uptake in CRC cells (Fig. 6D), indicating that lipid accumulation is at least partly driven by enhanced exogenous fatty acid influx rather than passive storage alone. DKK1 OE reduced lipid uptake, whereas GPx3 co-overexpression partially reversed this effect. Collectively, these results suggest that lipid accumulation in this model is associated with altered fatty acid uptake. Modulation of the GPx3–DKK1 axis regulates lipid influx, which is accompanied by changes in ferroptosis sensitivity.

To further validate these findings at the molecular level, we examined changes in ferroptosis-related biochemical markers. GSH is a key indicator of the cell’s antioxidant status. The result (Fig. 6E) showed that DKK1 OE significantly increased GSH levels in CRC cells, whereas DKK1 KD led to a decrease in GSH levels. When both GPx3 and DKK1 were overexpressed, GSH content was significantly lower than in the DKK1 OE group, which favored ferroptosis. The detection of lipid peroxidation product malondialdehyde (MDA) (Fig. 6F) showed that DKK1 OE cell had lower MDA levels compared to controls, whereas inhibiting DKK1 resulted in a significant increase in MDA content, indicating that DKK1 inhibits lipid peroxidation and reduces ferroptosis sensitivity. Remarkably, under the condition of dual knockdown of GPx3 and DKK1, MDA levels decreased compared to DKK1 KD alone. This suggests that GPx3 can weaken DKK1’s inhibitory effect on lipid peroxidation, thereby promoting an increase in ferroptosis sensitivity. Flow cytometric analysis of lipid peroxidation within cells (lipid ROS) further supported this conclusion (Fig. 6G).

To assess iron dependency, labile Fe^2+^ levels were measured using FerroOrange. Overexpression of GPx3 significantly increased Fe^2+^ levels, whereas GPx3 knockdown markedly reduced them, an effect that was reversed by concurrent silencing of DKK1 (Fig. S4). Collectively, these findings, together with the lipid ROS data, indicate that the GPx3–DKK1 axis sensitizes cells to ferroptosis by facilitating the accumulation of both catalytic iron and lipid peroxides. Cells overexpressing DKK1 exhibited lower lipid ROS levels, while dual overexpression of GPx3 and DKK1 significantly increased lipid ROS accumulation, indicating enhanced lipid peroxidation and increased ferroptosis sensitivity. Ultrastructural analysis was conducted using TEM to observe mitochondrial morphological changes under different treatments (Fig. 6H). The results showed that in cells with reduced DKK1 expression and elevated GPx3 levels, mitochondria exhibited reduced volume, rupture and collapse of the outer membrane, increased membrane density, and the disappearance of cristae. These features correspond to the characteristic mitochondrial changes observed during ferroptosis.

Finally, to clarify the molecular mechanism underlying the GPx3–DKK1 axis in ferroptosis sensitivity, we investigated the effects of this pathway on key ferroptosis regulatory factors. Prior work has identified DKK1 as an inhibitor of the Wnt signaling pathway and can suppress signal transducer and activator of transcription 3 (STAT3) activity, leading to up-regulation of the ferroptosis inhibitor SLC7A11, which decreases cellular lipid peroxidation and reduces ferroptosis sensitivity. Our Western blot experiments confirmed this mechanism. Increasing DKK1 expression significantly suppressed STAT3 levels in HCT-116 and SW480 cells, and this was accompanied by an up-regulation of SLC7A11 expression (Fig. 6I). This indicates that in CRC cells, DKK1 enhances SLC7A11 expression by inhibiting STAT3, thereby improving the cells’ antioxidant capacity and reducing ferroptosis sensitivity. In contrast, as shown (Fig. 6J), altering GPx3 expression did not significantly affect the level of another key ferroptosis enzyme, GPx4, but overexpression of GPx3 significantly down-regulated SLC7A11 expression, leading to reduced antioxidant capacity, enhanced lipid peroxidation, and increased sensitivity to ferroptosis. Collectively, these results indicate that elevated GPx3 and reduced DKK1 expression in CRC cells enhance ferroptosis sensitivity. Mechanistically, the GPx3–DKK1 axis modulates ferroptosis by regulating expression of the upstream key gene SLC7A11.

### In vivo role of the GPx3–DKK1 axis in CRC growth and metastasis

**Fig. 7.**
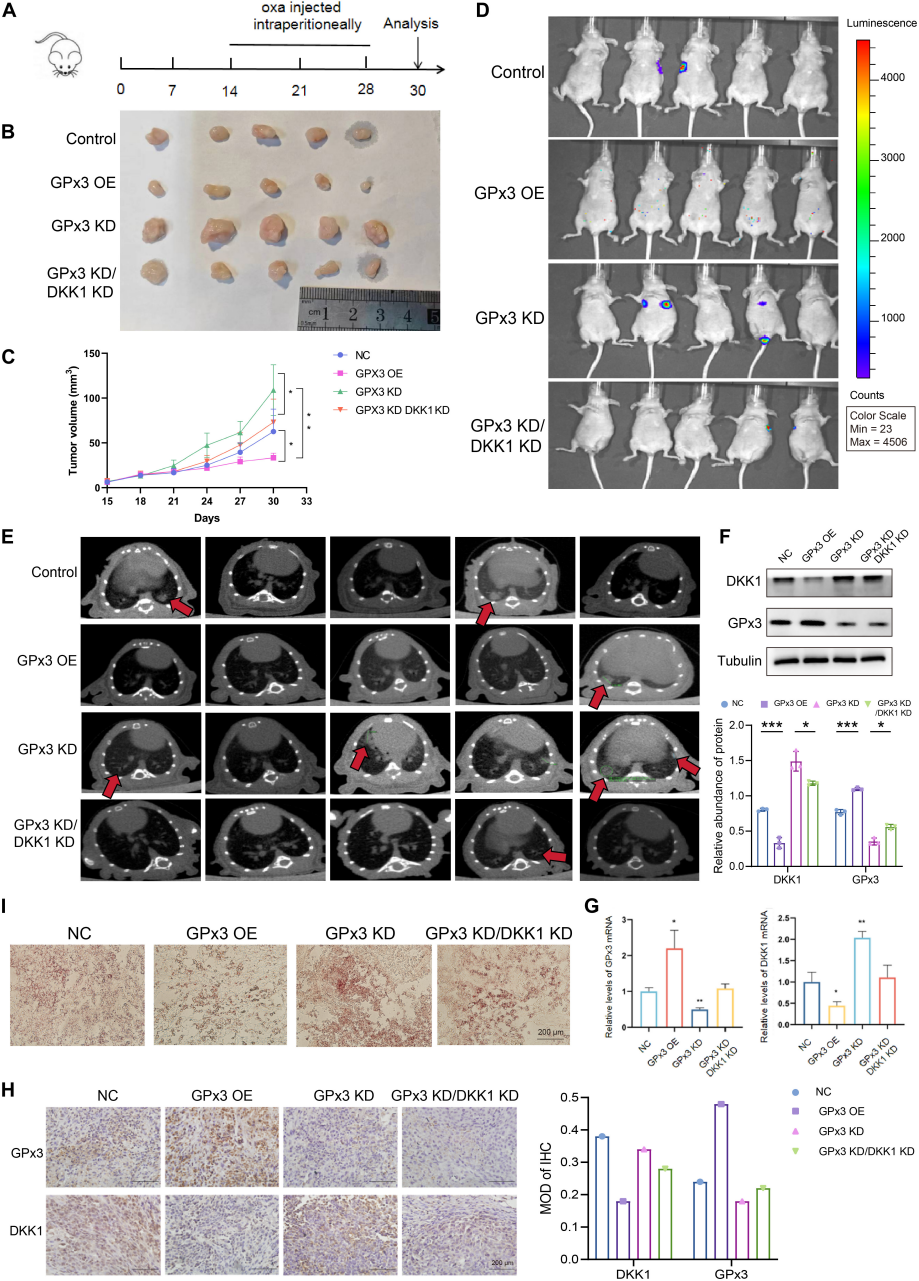
Glutathione peroxidase 3 (GPx3) inhibits colorectal cancer (CRC) growth and metastasis in vivo by regulating Dickkopf-1 (DKK1). (A) Schematic diagram of the experimental workflow and treatment schedule for the xenograft mouse model. Oxa, oxaliplatin. (B) Images of tumors dissected from each experimental group. (C) Tumor growth curves. (D and E) In vivo bioluminescence imaging and micro-computed-tomography scans showing lung metastasis. (F and G) Western blot and quantitative reverse transcription polymerase chain reaction (qRT-PCR) analysis of DKK1 and GPx3 expression in tumor tissues. (H) Representative immunohistochemical (IHC) staining images of GPx3 and DKK1 in tumor sections. (I) Oil Red O staining of tumor tissues showing lipid droplet (LD) accumulation. Data are presented as means ± SD. Statistical significance between 2 groups was determined by *t* test, and comparisons among multiple groups were performed using ANOVA analysis; *P < 0.05, **P < 0.01, and ***P < 0.001.

To assess the in vivo role of GPx3 in CRC progression, we established stable HCT-116 cell lines expressing GPx3 OE, GPx3 KD, or a double knockdown of GPx3 and DKK1 (GPx3 KD + DKK1 KD), alongside a negative control (NC). Cells were subcutaneously introduced into nude mice to form xenograft tumors (Fig. 7A). Tumor volume was monitored throughout the study, and oxaliplatin (8 mg/kg) was administered intraperitoneally every 4 d once tumors were palpable. After 24 d, tumors were excised and measured. GPx3 OE significantly reduced tumor volume and weight compared to the control, whereas GPx3 KD enhanced tumor growth. Notably, concurrent knockdown of DKK1 in the GPx3 KD background partially reversed this proliferative phenotype (Fig. 7B and C).

To assess metastatic potential, we injected luciferase-labeled derivatives of the same cell lines via the tail vein. After 6 weeks, in vivo imaging revealed that GPx3 OE strongly suppressed lung metastasis. In contrast, GPx3 KD increased metastatic burden, an effect that was again attenuated by coknockdown of DKK1 (Fig. 7D and E). Analysis of tumor tissues confirmed that GPx3 OE decreased, while GPx3 KD increased, DKK1 expression at both the mRNA and protein levels (Fig. 7F to H), consistent with GPx3-mediated transcriptional repression of DKK1. Furthermore, Oil Red O staining indicated a reduction in LD deposition in GPx3 OE tumors, whereas GPx3 KD enhanced lipid deposition—an effect reversed by simultaneous DKK1 KD (Fig. 7I). These in vivo results demonstrate that GPx3 constrains CRC growth and metastasis and that its suppressive effects are mediated, at least in part, through down-regulation of DKK1. Consistently, analysis of ferroptosis-associated markers, including SLC7A11, arachidonate 15-lipoxygenase (ALOX15), and Acyl-CoA synthetase long-chain family member 4 (ACSL4), further supported the involvement of the GPx3–DKK1 axis in ferroptosis regulation (Fig. S7). Although in vivo pharmacological inhibition of ferroptosis would provide additional causal validation, the coordinated regulation of canonical ferroptosis markers observed in tumor tissues supports a ferroptosis-associated mechanism underlying the GPx3/DKK1-dependent tumor phenotype. Targeting the GPx3–DKK1 axis may represent a promising therapeutic strategy in CRC.

### METTL3/14 down-regulates GPx3 expression via m^6^A methylation in CRC

**Fig. 8.**
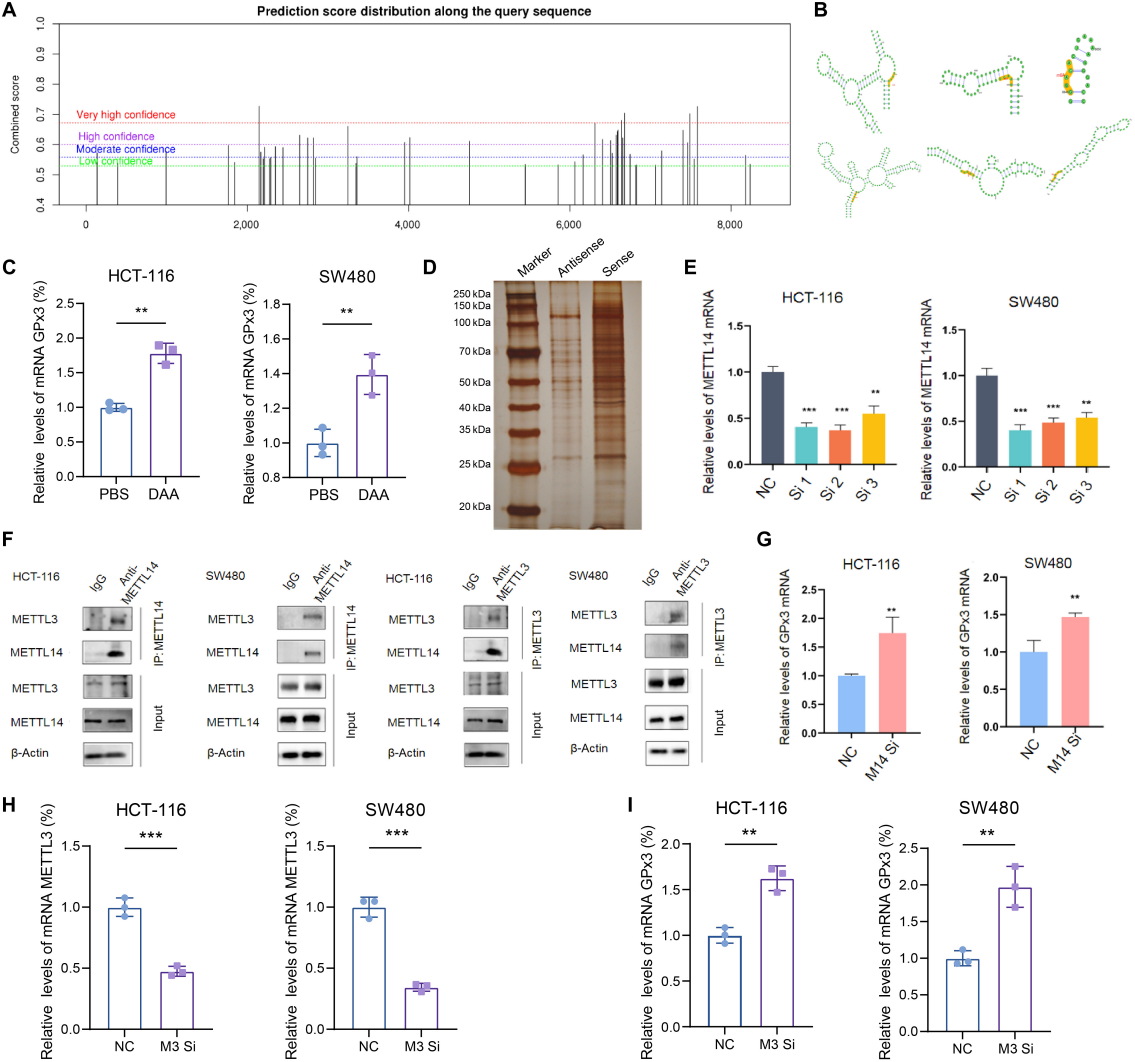
Methyltransferase-like protein 3/14 (METTL3/14) down-regulates glutathione peroxidase 3 (GPx3) expression via *N*^6^-methyladenosine (m^6^A) methylation. (A and B) Prediction of potential m^6^A modification sites and secondary structures on GPx3 mRNA using the SRAMP database. (C) Quantitative reverse transcription polymerase chain reaction (qRT-PCR) analysis of GPx3 expression after treatment with the m^6^A inhibitor 3-deazaadenosine (DAA). (D) Identification of proteins interacting with GPx3 mRNA by RNA pull-down, followed by mass spectrometry. (E and F) qRT-PCR analysis of METTL14 and GPx3 expression after METTL14 knockdown. IP, immunoprecipitation. (G) Coimmunoprecipitation assay confirming the interaction between METTL3 and METTL14 in colorectal cancer (CRC) cells. (H and I) qRT-PCR analysis of METTL3 and GPx3 expression after METTL3 knockdown. Data are presented as means ± SD; **P < 0.01 and ***P < 0.001 in unpaired *t* test.

To investigate the upstream mechanisms underlying GPx3 down-regulation in CRC, we first examined the baseline expression of m^6^A-related methyltransferases. Western blot analysis revealed that METTL14 protein expression was significantly reduced in CRC cells compared with the normal intestinal epithelial cell line NCM460 (Fig. S5). Given that residual m^6^A regulatory activity may remain functionally relevant despite reduced METTL14 expression, we next explored whether posttranscriptional m^6^A modification contributes to GPx3 repression in CRC. Bioinformatic analysis using the sequential reading and Multivariate model for m^6^A methylation site prediction (SRAMP) database predicted multiple putative m^6^A methylation sites within the GPx3 transcript (Fig. 8A and B), suggesting a potential role for RNA methylation in its regulation. Consistently, treatment of CRC cells with the m^6^A methylation inhibitor 3-deazaadenosine resulted in a marked increase in GPx3 expression (Fig. 8C), supporting the involvement of m^6^A modification in GPx3 suppression. To further validate whether the predicted m^6^A sites are functionally required for GPx3 regulation, we performed luciferase reporter assays using constructs containing either the wild-type (WT) or mutated (MUT) m^6^A consensus motifs within the GPx3 transcript. METTL3/14 overexpression significantly reduced luciferase activity in the WT construct, whereas this inhibitory effect was markedly attenuated in the MUT constructs (Fig. S6). These results indicate that m^6^A modification at the predicted sites is functionally involved in mediating GPx3 repression.

To identify m^6^A-related proteins that interact with GPx3 mRNA, we performed RNA pull-down assays, followed by mass spectrometry. METTL14 was significantly enriched among the bound proteins (Fig. 8D). Knockdown of METTL14 in SW480 and HCT-116 cells led to a pronounced up-regulation of GPx3, indicating its role in GPx3 repression (Fig. 8E and F). Given that METTL3 and METTL14 function as a complex in m^6^A methylation, with METTL14 recognizing substrate RNA and METTL3 catalyzing methyl transfer [52–54], we examined their interaction by coimmunoprecipitation. METTL3 coprecipitated with METTL14, and vice versa, confirming their direct binding in CRC cells (Fig. 8G). Furthermore, siRNA-mediated knockdown of METTL3 also increased GPx3 expression, consistent with the effect observed upon METTL14 depletion (Fig. 8H and I). Together, these findings indicate that GPx3 expression is suppressed in CRC through m^6^A methylation mediated by the METTL3/14 complex.

**Fig. 9.**
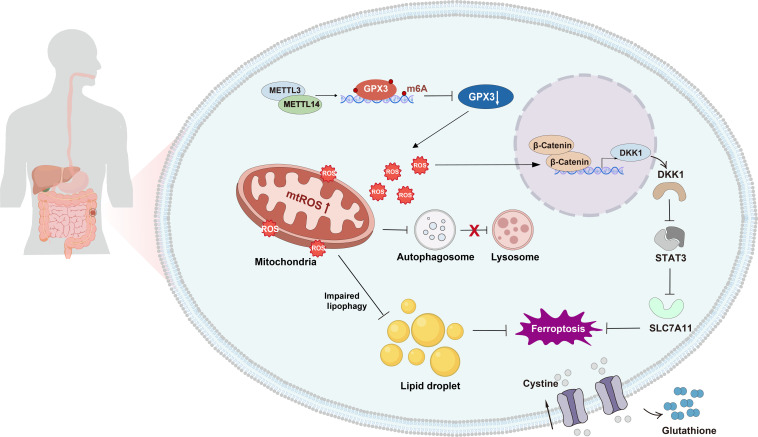
Schematic diagram of molecular mechanism proposed in this study.

In summary, m^6^A methylation suppresses GPx3 in CRC, elevating ROS and disrupting redox homeostasis. GPx3 loss increases mtROS and impairs LD lipophagy, causing lipid accumulation, reduced free fatty acids, and diminished ferroptosis sensitivity. Concomitantly, DKK1 is up-regulated, driving SLC7A11 expression, limiting lipid peroxidation and promoting malignant phenotypes. Targeting the METTL3/14–GPx3–DKK1–SLC7A11 axis may inhibit proliferation and metastasis while enhancing ferroptosis sensitivity, offering a potential therapeutic strategy for CRC (Fig. 9).

## Discussion

GPx3 appears to act as a tumor-suppressive factor in CRC. Prior studies in CRC models found that GPx3 loss promotes tumor formation and progression, suggesting its down-regulation contributes to malignancy [55,56]. In line with previous findings, we observed markedly lower GPx3 expression in both CRC tissues and cell lines relative to normal counterparts. Restoring GPx3 expression in CRC cells suppressed their malignant phenotypes, slowing proliferation and reducing migration and invasion in vitro. These findings support an important tumor-suppressive role of GPx3 in CRC.

Mechanistically, GPx3 appears to exert its tumor-suppressive effects partly by sustaining redox balance [29]. GPx3 is an antioxidant enzyme that scavenges ROS, and we found that GPx3 reexpression significantly reduced intracellular ROS levels, particularly mtROS, in CRC cells. Elevated mtROS can disrupt cell signaling and metabolic pathways to drive tumor cell proliferation and migration [57–59]. Thus, GPx3-mediated attenuation of mtROS is likely critical for restraining CRC progression. In GPx3-deficient CRC cells, conversely, excessive mtROS accumulates and instigates downstream effects on cellular metabolism and survival pathways.

One major consequence of mtROS accumulation in GPx3-silenced cells is impaired lipophagy. Excessive mtROS interferes with autophagosome–lysosome fusion, hindering the autophagic flux that normally clears LDs [60]. Consistent with this, we observed that GPx3 down-regulation led to abnormal LD accumulation in CRC cells: TEM revealed a build-up of cytosolic LDs accompanied by a marked reduction in autophagosomes containing lipid cargo. These results indicate that loss of GPx3 disrupts LD turnover, causing an overload of lipid stores within the cells.

Abnormal accumulation of LDs is a hallmark of tumor metabolic reprogramming [61]. Sequestering neutral lipids in droplets provides cancer cells with an energy reservoir and essential membrane components, while also curbing lipotoxicity. Notably, by sequestering fatty acids in stored form, LDs reduce the pool of free fatty acids available for lipid peroxidation. This is significant because peroxidation of polyunsaturated fatty acids is a trigger for ferroptosis, a cell death mode driven by iron dependence [62,63]. In GPx3-deficient cells, the increased LD content may arise from multiple metabolic routes, including enhanced exogenous fatty acid uptake as well as impaired lipid catabolic degradation rather than enhanced lipid synthesis, thereby sustaining lipid sequestration and limiting lipid peroxidation [64]. This protection enhances the cells’ ability to survive under stress, promotes malignant proliferation and metastasis, and can even reduce the effectiveness of chemotherapy, ultimately facilitating therapy resistance. In summary, GPx3 loss-induced LD accumulation contributes to an intrinsic resistance to ferroptosis and supports tumor aggressiveness.

In addition to the lipophagy defect, we identified a complementary pathway by which GPx3 down-regulation fosters ferroptosis resistance. Transcriptomic and proteomic analyses of GPx3 KD CRC cells revealed a significant up-regulation of DKK1, a Wnt signaling inhibitor. DKK1 is frequently overexpressed in CRC and is known to promote tumor cell proliferation and migration [65,66]. Importantly, DKK1 can drive the expression of the cystine–glutamate antiporter SLC7A11, increasing intracellular GSH synthesis and protecting cells from lipid peroxidation damage, thereby enhancing resistance to ferroptosis [26,67]. It is noteworthy that HCT-116 and SW480 cells differ in p53 status, a well-established regulator of ferroptosis through transcriptional repression of SLC7A11. Despite this difference, GPx3 modulation induced concordant changes in SLC7A11 expression in both cell lines, indicating that the GPx3–DKK1–SLC7A11 axis operates largely independently of p53 status, although p53 may quantitatively modulate ferroptosis sensitivity in a context-dependent manner. Our findings indicate that elevated mtROS in GPx3-deficient cells may activate this DKK1–SLC7A11 axis. In GPx3-low CRC cells, the mtROS surge is accompanied by DKK1 up-regulation and subsequent induction of SLC7A11, leading to higher GSH levels and suppression of ferroptotic cell death. Through this mechanism, loss of GPx3 triggers a signaling cascade that reinforces the ferroptosis-resistant state of the cancer cells, further promoting their malignant progression.

To delineate the mechanisms underlying GPx3 loss in CRC, we investigated epigenetic regulation with a particular focus on m^6^A, a prevalent posttranscriptional modification implicated in tumorigenesis. Bioinformatic analyses predicted multiple putative m^6^A sites within the GPx3 transcript. Consistently, RNA pull-down followed by mass spectrometry identified METTL3/METTL14 as GPx3-associated methyltransferases, and coimmunoprecipitation assays confirmed m^6^A modification on GPx3 mRNA. Functionally, METTL3/14-dependent m^6^A deposition correlated with reduced GPx3 protein abundance in CRC cells. Notably, although METTL14 is globally down-regulated and widely regarded as a tumor suppressor in CRC, our findings indicate that residual METTL3/14 activity remains functionally relevant and exerts transcript-specific regulatory effects. In this context, m^6^A-mediated repression of GPx3 represents a target-selective mechanism contributing to redox imbalance and tumor progression, rather than a contradiction of METTL14’s established tumor-suppressive role. Nonetheless, locus-specific m^6^A editing strategies and in vivo validation will be required to establish direct causality and evaluate the therapeutic tractability of this regulatory axis.

Integrating these observations, we propose a hierarchically organized but functionally branched model centered on GPx3. In this framework, METTL3/14-mediated m^6^A modification acts as an upstream regulatory event leading to GPx3 down-regulation, while GPx3 serves as a central node controlling mtROS homeostasis. Elevated mtROS subsequently drives 2 interconnected but partially independent downstream processes: impaired lipophagy resulting in LD accumulation and activation of the β-catenin–DKK1–SLC7A11 axis, which suppresses ferroptosis sensitivity. Together, these pathways converge to promote metabolic reprogramming and ferroptosis resistance in CRC. These findings refine the mechanistic link between redox regulation and lipid metabolic homeostasis in CRC. Targeting the GPx3-centered regulatory network, including both the mtROS–lipophagy axis and the DKK1–SLC7A11 pathway, may provide potential therapeutic strategies to inhibit tumor progression and enhance ferroptosis-based treatments.

## Conclusion

This study shows that GPx3 down-regulation in colon cancer is linked to redox imbalance, impaired lipophagy, and an antiferroptotic program involving the DKK1–SLC7A11 axis, consistent with enhanced tumor cell survival, migration, and therapeutic resistance. These findings suggest a testable m^6^A–GPx3–lipid–metabolism/antiferroptosis regulatory axis and provide a rationale to assess GPx3 restoration or modulation of ferroptosis pathways as potential interventions.

## Methods

### Clinical data and human samples

CRC specimens were procured from patients at Tianjin Medical University Cancer Institute and Hospital who possessed comprehensive clinical records. From these participants, we collected paired fresh tumor and adjacent nontumor tissues. The study protocol received ethical clearance from the Ethics Committee of Tianjin Medical University Cancer Hospital (approval no. EK20250289). Furthermore, written informed consent regarding the utilization of tissue samples was secured from every participant [68].

### Animals

Female BALB/c-nu and C57BL/6 mice were purchased from Jiangsu Jicui Pharmachem Biotechnology. These animals were maintained in a specific-pathogen-free facility with water and standard chow available ad libitum. All animal experimental procedures were authorized by the Institutional Animal Care and Research Advisory Committee of TMUCIH (protocol no. NSFC-AE-2025394). [69].

### Cell lines and cell culture

NCM460 (RRID: CVCL_0460), a normal human colonic epithelial cell line, was utilized in this study alongside 2 CRC cell lines: HCT-116 (RRID: CVCL_0291) and SW480 (RRID: CVCL_0546). All cell lines were purchased from the Cell Bank of the Shanghai Institute of Life Sciences (China) and were subsequently passaged and cryopreserved in our laboratory. All cell lines were authenticated by short tandem repeat profiling and routinely tested for mycoplasma contamination with negative results. We confirmed the absence of mycoplasma contamination in all cell lines. NCM460, HCT-116, and SW480 cells were grown in RPMI 1640 basal medium enriched with 10% fetal bovine serum and 1% penicillin/streptomycin. All cell culture reagents were purchased from Gibco (Beijing, China). The cultures were incubated at 37 °C in a humidified atmosphere containing 5% CO_2_ [70].

### Western blotting

Total protein lysates were harvested from cell or tissue samples utilizing radioimmunoprecipitation assay buffer supplemented with 1% phenylmethylsulfonyl fluoride (PMSF; Solarbio, Beijing, China). Protein yields were quantified via a bicinchoninic acid (BCA) assay kit (Thermo Fisher Scientific, Waltham, MA, USA). Following heat denaturation at 95 °C in loading buffer, the samples were resolved by sodium dodecyl sulfate–polyacrylamide gel electrophoresis and subsequently electrotransferred onto Roche polyvinylidene difluoride membranes. We blocked nonspecific binding using 5% nonfat dry milk (Solarbio). The blots were then probed with primary antibodies overnight at 4 °C, succeeded by incubation with horseradish-peroxidase-linked secondary antibodies (Solarbio). Specific proteins were detected using an enhanced chemiluminescence kit. Antibodies included GPx3 (1:1,000; Abcam, ab256470), DKK1 (1:1,000; Proteintech, 21112-1-AP), SLC7A11 (1:1,000; Abcam, ab307601), GPx4 (1:1,000; Proteintech, 67763-1-Ig), β-catenin (1:1,000; Proteintech, 51067-2-AP), E-cadherin (1:1,000; Abcam, ab314063), Snail (1:1,000; Proteintech, 12129-1-AP), tubulin (1:5,000; Santa Cruz Biotechnology, sc-73242), glyceraldehyde-3-phosphate dehydrogenase (GAPDH; 1:200; Santa Cruz Biotechnology, sc-137179), β-actin (1:200; Santa Cruz Biotechnology, sc-58673), and Vimentin (1:1,000; Abcam, ab92547).

### Cell transfection

Cells were seeded at a density of 2 × 10^5^ to 3 × 10^5^ cells per well (6-well plate) 1 d prior to transfection. Before transfection, the medium was replaced with 1.5 ml of serum-free basal medium (Gibco, Beijing, China). Transfection complexes were prepared using Lipofectamine 2000 (Invitrogen, Carlsbad, CA, USA) and siRNA (100 pmol per well) (RiboBio, Guangzhou, China) or plasmid (4 μg per well) (TIANGEN) diluted in Opti-MEM (Gibco, Beijing, China), incubated for 20 min at room temperature, and then added to the cells. After 4 to 6 h, the medium was replaced with fresh complete RPMI 1640. Cells were harvested for RNA (TIANGEN) after 24 h and for protein after 48 h [71].

### Lentiviral infection

Lentiviral transduction was performed in the presence of polybrene (5 to 10 μg/ml; Jikai Gene Chemical Technology Co. Ltd., Shanghai, China) to enhance efficiency. Following an incubation period of 8 to 12 h, the virus-laden medium was discarded and replaced with fresh complete medium. Antibiotic selection commenced 72 h posttransduction using puromycin (2 μg/ml; Solarbio, Beijing, China) and was maintained for a duration of 2 weeks. The establishment of stable cell lines was subsequently verified by RNA and protein assays.

### Colony formation assay

For the colony formation assay, cells were inoculated into 6-well plates at a density of 500 cells per well and allowed to grow for 14 d. Upon completion of the culture period, colonies were immobilized using 4% paraformaldehyde and visualized by staining with crystal violet (Solarbio, Beijing, China). Colony counts were determined from captured images.

### EdU proliferation assay

Cells were incubated with 50 μM EdU for 12 h, fixed, permeabilized with 0.2% Triton X-100, and stained using the Cell-Light EdU Apollo Stain Kit (RiboBio, Guangzhou, China) according to the manufacturer’s instructions. All steps were performed protected from light.

### CCK-8 assay for IC_50_ determination

Cells were plated at a density of 5,000 cells per well in 96-well plates and exposed to varying concentrations of the drug gradients. Following the treatment period, CCK-8 reagent (Solarbio, Beijing, China) was introduced to each well. Absorbance was subsequently recorded at 450 nm. The half-maximal inhibitory concentration (IC_50_) was computed using GraphPad Prism software.

### Wound healing assay

A linear scratch was created across confluent cell monolayers utilizing a sterile pipette tip. The wound closure process was monitored and photographed at specific time intervals: 0, 12, 24, and 48 h. The extent of cell migration was analyzed via ImageJ software.

### Transwell migration assay

Cells underwent serum starvation before being inoculated into the upper compartment of Transwell inserts (Corning). The lower reservoir was filled with medium supplemented with 20% fetal bovine serum to serve as a chemoattractant. Following a 24-h incubation, cells that had traversed the membrane were fixed, subjected to crystal violet staining, and enumerated.

### Immunofluorescence

Cells grown on coverslips were fixed, permeabilized, blocked with 2% bovine serum albumin, and incubated with primary antibodies at 4 °C overnight. After incubation with fluorescent secondary antibodies, nuclei were stained with 4′,6-diamidino-2-phenylindole (DAPI). Coverslips were mounted and imaged using a fluorescence microscope [72].

### Fe^2+^ levels analysis

Cells were treated according to the experimental design and incubated with FerroOrange fluorescent probe (Dojindo, Japan) at 37 °C in the dark for 30 min. Fluorescence signals were then detected and captured using a fluorescence microscope under the appropriate excitation wavelength. The fluorescence intensity was used to reflect intracellular labile Fe^2+^ levels.

### Luciferase reporter assay

The WT GPx3 3′ untranslated region and MUT m^6^A were cloned into the pmiR-RB-REPORT vector (RiboBio). The reporter constructs were cotransfected with METTL3/14 expression plasmids into HCT-116 and SW480 cells using Lipofectamine 3000. After 48 h of transfection, luciferase activity was measured using the Dual-Luciferase Reporter Assay System (Promega, USA).

### Chromatin immunoprecipitation

Cells were cross-linked with 1% formaldehyde for 10 min at room temperature, followed by quenching with 0.125 M glycine. After washing with phosphate-buffered saline (PBS), cells were lysed in PMSF-containing lysis buffer. Chromatin was sonicated to fragments of 200 to 1,000 bp. Immunoprecipitation was performed overnight at 4 °C using 2 μg of target-specific antibody or control IgG, followed by incubation with magnetic beads (Selleck Chemicals). Complexes were washed sequentially with low-salt, high-salt, and LiCl wash buffers. Elution was carried out using ChIP elution buffer containing 1% Proteinase K. After reverse cross-linking, DNA was purified and analyzed by qPCR (as described above). Primer sequences are provided in Plasmid Information in the Supplementary Materials.

### RNA pull-down assay

Cells were digested with 0.05% trypsin, centrifuged at 1,000 ×g for 3 min, and washed twice with ice-cold PBS. The cell pellet was lysed in lysis buffer supplemented with PMSF and protease inhibitor cocktail, followed by centrifugation at 12,000*g* for 10 min at 4 °C. The supernatant was collected and stored at −80 °C. Streptavidin magnetic beads were washed and resuspended in RNA capture buffer and then incubated with biotin-labeled RNA for 30 min. After washing, the RNA-bound beads were resuspended in 1× protein–RNA binding buffer and incubated with cell lysate (30 μg of protein) for 1 h at 4 °C with gentle rotation. The beads were washed 5 times, and bound proteins were eluted using biotin elution buffer for downstream mass spectrometry analysis [73].

### Establishment of mouse xenograft tumor models

Female BALB/c-nu mice (4 weeks of age) were randomized into 4 cohorts (*n* = 5 per group). Each mouse received a subcutaneous injection of 5 × 10^6^ cells suspended in 100 μl of PBS into the inguinal region. Tumor dimensions were assessed on a weekly basis. Starting 7 d postinoculation, Oxaliplatin (8 mg/kg) was delivered via intraperitoneal injection every 4 d. Upon conclusion of the study (1 month), the animals were euthanized, and tumor tissues were harvested for subsequent weight measurement, photography, and molecular assays.

### Metastasis model

Luciferase-labeled HCT-116 cells with modulated expression of GPx3 and/or DKK1 were constructed. Mice were injected via the tail vein with 1 × 10^6^ cells in 100 μl of PBS. Lung metastasis was monitored using in vivo bioluminescence imaging and microcomputed tomography 3 to 4 weeks after injection.

### Oil Red O staining

To detect neutral lipids, cryosectioned tumor samples (6 to 10 μm) were fixed and incubated with the Oil Red O working solution for 15 min at room temperature. Destaining was performed in 60% isopropanol. After washing, the sections were sealed and photographed. Neutral lipids were visualized as dark orange–red deposits [74,75].

### IHC staining

Tissue samples were fixed in paraformaldehyde, embedded in paraffin, and sectioned at 3 to 7 μm in thickness. Sections were deparaffinized in xylene and rehydrated through a graded ethanol series. Antigen retrieval was performed in citrate or EDTA buffer (pH 6.0 or 9.0) using a pressurized heating method. After blocking with 3% H_2_O_2_, sections were incubated with primary antibodies at 4 °C overnight, followed by incubation with horseradish-peroxidase-conjugated secondary antibodies. Signal was developed using 3,3′-diaminobenzidine (DAB), and nuclei were counterstained with hematoxylin. Sections were dehydrated, cleared in xylene, and mounted with neutral balsam. Images were acquired from 5 randomly selected fields per sample [74].

### Lipid ROS level detection

Prior to staining, cells were rinsed with serum-free medium. Subsequently, they were exposed to 10 μM BODIPY 581/591 C11 (Thermo Fisher Scientific, Waltham, MA, USA) and incubated at 37 °C for 30 min in the dark. Following this, the cells were washed, harvested using trypsin, and resuspended in PBS for subsequent analysis via flow cytometry [76,77].

### Statistical analysis

All experiments were performed in at least triplicate, and results are presented as the means ± standard deviation (SD). Statistical analyses were carried out in SPSS, using Student’s *t* test for 2-group comparisons and analysis of variance (ANOVA) for analyses involving multiple groups. A *P* < 0.05 was considered statistically significant.

## Ethical Approval

The collection of patient tissues was approved by the Ethics Committee of Tianjin Medical University Cancer Hospital (approval ID: EK20250289). The animal trials received approval from the Institutional Animal Care and Research Advisory Committee of TMUCIH (NSFC-AE-2025394).

## Data Availability

The datasets used and/or analyzed in the current study are available from the corresponding authors on reasonable request.

## Supplementary Materials

Supplementary 1 Figs. S1 to S7 Uncropped Western Blot Plasmid Information List of Abbreviations
