# Supplementary material for: Methyltransferase-like 3/14-mediated m6A Silencing of GPx3 Drives Lipophagy Dysfunction and Ferroptosis Resistance in Colorectal Cancer
Source: Research (Wash D C). 2026 May 11;9:1273. doi: 10.34133/research.1273 (PMC13158459; doi:10.34133/research.1273)
Supplement: Supplementary 1 — Figs. S1 to S7 Uncropped Western Blot Plasmid Information List of Abbreviations [file research.1273.f1.zip › List of abbreviations.pdf]

Abbreviation	Full name
ACC	Adrenocortical Carcinoma
BLCA	Bladder Urothelial Carcinoma
BRCA	Breast Invasive Carcinoma
CESC	Cervical Squamous Cell Carcinoma and Endocervical Adenocarcinoma
CHOL	Cholangiocarcinoma
COAD	Colon Adenocarcinoma
DLBC	Lymphoid Neoplasm Diffuse Large B-cell Lymphoma
ESCA	Esophageal Carcinoma
GBM	Glioblastoma Multiforme
HNSC	Head and Neck Squamous Cell Carcinoma
KICH	Kidney Chromophobe
KIRC	Kidney Renal Clear Cell Carcinoma
KIRP	Kidney Renal Papillary Cell Carcinoma
LAML	Acute Myeloid Leukemia
LGG	Brain Lower Grade Glioma
LIHC	Liver Hepatocellular Carcinoma
LUAD	Lung Adenocarcinoma
LUSC	Lung Squamous Cell Carcinoma
MESO	Mesothelioma
OV	Ovarian Serous Cystadenocarcinoma
PAAD	Pancreatic Adenocarcinoma
PCPG	Pheochromocytoma and Paraganglioma
PRAD	Prostate Adenocarcinoma

READ	Rectum Adenocarcinoma
SARC	Sarcoma
SKCM	Skin Cutaneous Melanoma
STAD	Stomach Adenocarcinoma
TGCT	Testicular Germ Cell Tumors
THCA	Thyroid Carcinoma
THYM	Thymoma
UCEC	Uterine Corpus Endometrial Carcinoma
UCS	Uterine Carcinosarcoma
UVM	Uveal Melanoma
