# Supplementary material for: Methyltransferase-like 3/14-mediated m6A Silencing of GPx3 Drives Lipophagy Dysfunction and Ferroptosis Resistance in Colorectal Cancer
Source: Research (Wash D C). 2026 May 11;9:1273. doi: 10.34133/research.1273 (PMC13158459; doi:10.34133/research.1273)
Supplement: Supplementary 1 — Figs. S1 to S7 Uncropped Western Blot Plasmid Information List of Abbreviations [file research.1273.f1.zip › Plasmid Information.pdf]

GPX3 OE

GGTGTCCACTCCCAGGTCCAACTGCACCTCGGTTCTAAGCTTCTGCAGGTCGACTCTAGAGGATCCCGCCAC
CATGGCCCGGCTGCTGCAGGCGTCCTGCCTGCTTTCCCTGCTCCTGGCCGGCTTCGTCTCGCAGAGCCGGG
GACAAGAGAAGTCGAAGATGGACTGCCATGGTGGCATAAGTGGCACCATTTACGAGTACGGAGCCCTCACC
ATTGATGGGGAGGAGTACATCCCCTTCAAGCAGTATGCTGGCAAATACGTCCTCTTTGTCAACGTGGCCAGC
TACTGAGGCCTGACGGGCCAGTACATTGAACTGAATGCACTACAGGAAGAGCTTGCACCATTCGGTCTGGT
CATTCTGGGCTTTCCCTGCAACCAATTTGGAAAACAGGAACCAGGAGAGAACTCAGAGATCCTTCCTACCCT
CAAGTATGTCCGACCAGGTGGAGGCTTTGTCCCTAATTTCCAGCTCTTTGAGAAAGGGGATGTCAATGGAG
AGAAAGAGCAGAAATTCTACACTTTCCTAAAGAACTCCTGTCCTCCCACCTCGGAGCTCCTGGGTACATCTG
ACCGCCTCTTCTGGGAACCCATGAAGGTTCACGACATCCGCTGGAACTTTGAGAAGTTCCTGGTGGGGCCA
GATGGTATACCCATCATGCGCTGGCACCACCGGACCACGGTCAGCAACGTCAAGATGGACATCCTGTCCTAC
ATGAGGCGGCAGGCAGCCCTGGGGGTCAAGAGGAAGTAACTGAAGGCCGTCTCATCCCATGTCCACCATGT
AGGGGAGGGACTTTGTTCAGGAAGAAATCCGTGTCTCCAACCACACTATCTACCCATCACAGACCCCTTTCC
TATCACTCAAGGCCCCAGCCTGGCACAAATGGATGCATACAGTTCTGTGTACTGCCAGGCATGTGGGTGTGG
GTGCATGTGGGTGTTTACACACATGCCTACAGGTATGCGTGATTGTGTGTGTGTGCATGGGTGTACAGCCAC
GTGTCTACCTATGTGTCTTTCTGGGAATGTGTACCATCTGTGTGCCTGCAGCTGTGTAGTGCTGGACAGTGAC
AACCCTTTCTCTCCAGTTCTCCACTCCAATGATAATAGTTCACTTACACCTAAACCCAAAGGAAAAACCAGCT
CTAGGTCCAATTGTTCTGCTCTAACTGATACCTCAACCTTGGGGCCAGCATCTCCCACTGCCTCCAAATATTAG
TAACTATGACTGACGTCCCCAGAAGTTTCTGGGTCTACCACACTCCCCAACCCCCCACTCCTACTTCCTGAAG
GGCCCTCCCAAGGCTACATCCCCACCCCACAGTTCTCCCTGAGAGAGATCAACCTCCCTGAGATCAACCAAG
GCAGATGTGACAGCAAGGGCCACGGACCCCATGGCAGGGGTGGCGTCTTCATGAGGGAGGGGCCCAAAG
CCCTTGTGGGCGGACCTCCCCTGAGCCTGTCTGAGGGGCCAGCCCTTAGTGCATTCAGGCTAAGGCCCCTG
GGCAGGGATGCCACCCCTGCTCCTTCGGAGGACGTGCCCTCACCCCTCACTGGTCCACTGGCTTGAGACTC
ACCCCGTCTGCCCAGTAAAAGCCTTTCTGCAGCACTCGAGTCCATCGATACTAGTCTGTGGAATGTGTGTCAG
TTAGGGTGTGGAAAGTCCCCAGGCTCCCCAGCAGGCAGAAGTATGCAAAGCATGCATCTCAATTAGTCAGC
AACCAGGTGTGGAAAGTCCCCAGGCTCCCCAGCAGGCAGAAGTATGCAAAGCATGCATCTCAATTAGTCAG
CAACCATAGTCCCGCCCCTAACTCCGCCCATCCCGCCCCTAACTCCGCCCAGTTCCGCCCATTCTCCGCCCCAT
GGCTGACTAATTTTTTTTATTTATGCAGAGGCCGAGGCCGCCTCTGCCTCTGAGCTATTCCAGAAGTAGTGA
GGAGGCTTTTTTGGAGGCCTAGGCTTTTGCAAAAAGCTCCCGGGAGCTTGTATATCCATTTTCGGATCTGAT
CGCCACCATGACCGAGTACAAGCCCACGGTGCGCCTCGCCACCCGCGACGACGTCCCCAGGGCCGTACGCA
CCCTCGCCGCCGCGTTCGCCGACTACCCCGCCACGCGCCACACCGTCGATCCGGACCGCCACATCGAGCGG
GT

GPX3 KD

GGCAATCAACTAAAGATTACAAAAACAAATTACAAAAATTCAAAATTTTCGGGTTTATTACAGGGACAGCAGAGATCCA
GTTTGGTTAGTACCGGGCCCGCTCTAGACTCGAGCGGCCGCCCCCTTCACCGAGGGCCTATTTCCCATGATTCCTTCAT
ATTTGCATATACGATACAAGGCTGTTAGAGAGATAATTGGAATTAATTTGACTGTAAACACAAAGATATTAGTACAAAA
TACGTGACGTAGAAAGTAATAATTTCTTGGGTAGTTTGCAGTTTTAAAATTATGTTTTAAAATGGACTATCATATGCTT
ACCGTAACTTGAAAGTATTTCGATTTCTTGGCTTTATATATCTTGTGGAAAGGACGAAACACCGGGTGGAGGCTTTGTC
CCTAATTCTCGAGAATTAGGGACAAAGCCTCCACTTTTTGAATTCGGATCCATTAGGCGGCCGCGTGGATAACCGTATT
ACCGCCATGCATTAGTTATTAATAGTAATCAATTACGGGGTCATTAGTTCATAGCCCATATATGGAGTTCCGCGTTACA
TAACTTACGGTAAATGGCCCGCCTGGCTGACCGCCCAACGACCCCCGCCCATTGACGTCAATAATGACGTATGTTCCCA
TAGTAACGCCAATAGGGACTTTCCATTGACGTCAATGTGTGGAGTATTTACGGTAAACTGCCCACTTGGCAGTACATCA
AGTGTATCATATGCCAAGTACGCCCCCTATTGACGTCAATGACGGTAAATGGCCCGCCTGGCATTATGCCCAGTACATG
ACCTTATGGGACTTTCCTACTTGGCAGTACATCTACGTATTAGTCATCGCTATTACCATGCTGATGCGGGTTTGGCAGT
ACATCAATGGGCGTGGATAGCGGTTTTGACTCACGGGGATTTCCAAGTCTCCACCCCCATTTGACGTCATGGGAGTTTG
TTTTGGCACCAAAATCAACGGGACTTTTCCAAAATTGTCCGTAACCAACCTC

PSC116908-1 ccggGTGGAGGCTTTGTCCCTAATTctcgagAATTAGGGACAAAGCCTCCACtttttg

DKK1 OE

TTTTGGCTTTTTTGTTAGACGAAGCTTGGGCTGCAGGTCGACTCTAGAGGATCCAACTTTGTGCCAACCGGT
CGCCACCATGATGGCTCTGGGCGCAGCGGGAGCTACCCGGGTCTTTGTCGCGATGGTAGCGGCGGCTCTCG
GCGGCCACCCTCTGCTGGGAGTGAGCGCCACCTTGAACTCGGTTCTCAATTCCAACGCTATCAAGAACCTGC
CCCCACCGCTGGGCGGCGCTGCGGGGCACCCAGGCTCTGCAGTCAGCGCCGCGCCGGGAATCCTGTACCC
GGGCGGGAATAAGTACCAGACCATTGACAACTACCAGCCGTACCCGTGCGCAGAGGACGAGGAGTGCGGC
ACTGATGAGTACTGCGCTAGTCCCACCCGCGGAGGGGACGCAGGCGTGCAAATCTGTCTCGCCTGCAGGAA
GCGCCGAAAACGCTGCATGCGTCACGCTATGTGCTGCCCCGGGAATTACTGCAAAAATGGAATATGTGTGTC
TTCTGATCAAAATCATTTCCGAGGAGAAATTGAGGAAACCATCACTGAAAGCTTTGGTAATGATCATAGCAC
CTTGGATGGGTATTCCAGAAGAACCACCTTGTCTTCAAAAATGTATCACACCAAAGGACAAGAAGGTTCTGT
TTGTCTCCGGTCATCAGACTGTGCCTCAGGATTGTGTTGTGCTAGACACTTCTGGTCCAAGATCTGTAAACCT
GTCCTGAAAGAAGGTCAAGTGTGTACCAAGCATAGGAGAAAAGGCTCTCATGGACTAGAAATATTCCAGCG
TTGTTACTGTGGAGAAGGTCTGTCTTGCCGGATACAGAAAGATCACCATCAAGCCAGTAATTCTTCTAGGCTT
CACACTTGTCAGAGACACAAGCTCAGAGTTGGCATTGACTACAAGGATGACGATGACAAGGATTACAAAGA
CGACGATGATAAGGACTATAAGGATGATGACGACAAATAAGAATTCCTGTG

DKK1 KD

AGAATTCAACTAAAAGATTACAAAAACAAATTACAAAAATTCAAAATTTTCGGGTTTATTACAGGGACAGCAGAGATCC
AGTTTGGTTAGTACCGGGCCCGCTCTAGACTCGAGCGGCCGCCCCCTTCACCGAGGGCCTATTTCCCATGATTCCTTCA
TATTTGCATATACGATACAAGGCTGTTAGAGAGATAATTGGAATTAATTTGACTGTAAACACAAAGATATTAGTACAAA
ATACGTGACGTAGAAAGTAATAATTTCTTGGGTAGTTTGCAGTTTTAAAATTATGTTTTAAAATGGACTATCATATGCT
TACCGTAACTTGAAAGTATTTCGATTTCTTGGCTTTATATATCTTGTGGAAAGGACGAAACACCGGCCTGTCCTGAAAG
AAGGTCAACTCGAGTTGACCTTCTTTCAGGACAGGTTTTTGAATTCGGATCCATTAGGCGGCCGCGTGGATAACCGTAT
TACCGCCATGCATTAGTTATTAATAGTAATCAATTACGGGGTCATTAGTTCATAGCCCATATATGGAGTTCCGCGTTAC
ATAACTTACGGTAAATGGCCCGCCTGGCTGACCGCCCAACGACCCCCGCCCATTGACGTCAATAATGACGTATGTTCCC
ATAGTAACGCCAATAGGGACTTTCCATTGACGTCAATGGGTGGAGTATTTACGGTAAACTGCCCACTTGGCAGTACATC
AAGTGTATCATATGCCAAGTACGCCCCCTATTGACGTCAATGACGGTAAATGGCCCGCCTGGCATTATGCCCAGTACAT
GACCTTATGGGACTTTCCTACTTGGCAGTACATCTACGTATTAGTCATCGCTATTACCATGGTGATGCGGTTTTGGCAG
TACATCAATGGGCGTGGATAGCGGGTTTGACTCACGGGGATTTCCAAGTCTCCACCCCATTGACGTCAATGGGAGTTTG
TTTTGGCACAAAATCAACGGGACTTTCCAAATGTCGTACAACTCCGCCCCAT

PSC118424-1 ccggCCTGTCCTGAAAGAAGGTCAActcgagTTGACCTTCTTTCAGGACAGGtttttg
