# Supplementary material for: Methyltransferase-like 3/14-mediated m6A Silencing of GPx3 Drives Lipophagy Dysfunction and Ferroptosis Resistance in Colorectal Cancer
Source: Research (Wash D C). 2026 May 11;9:1273. doi: 10.34133/research.1273 (PMC13158459; doi:10.34133/research.1273)

FIG1F

GPx3

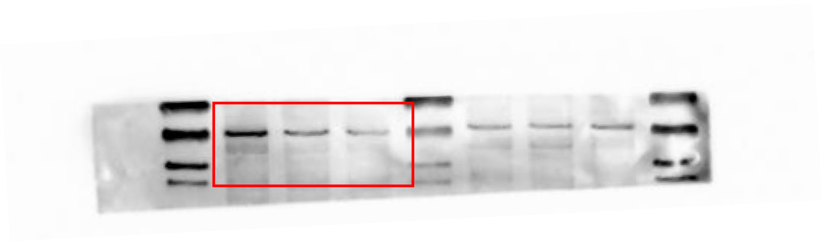

GAPDH

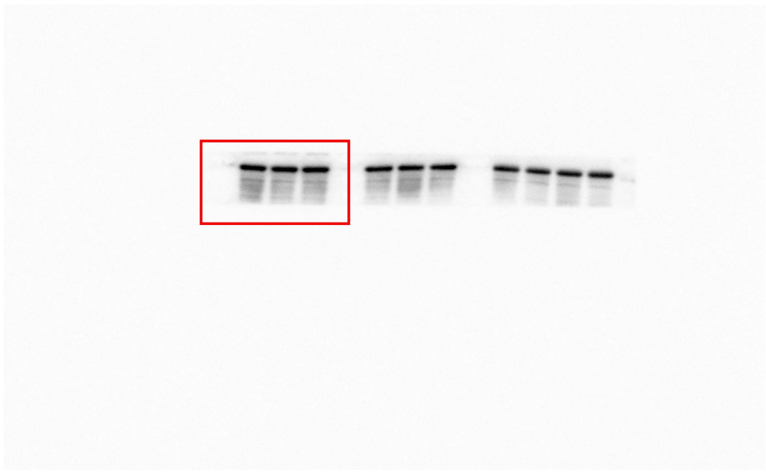

Fig2B

HCT-116 GPx3

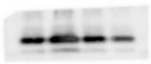

SW480 GPx3

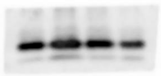

HCT-116 tubulin

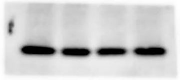

SW480 tubulin

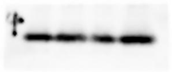

Fig2G

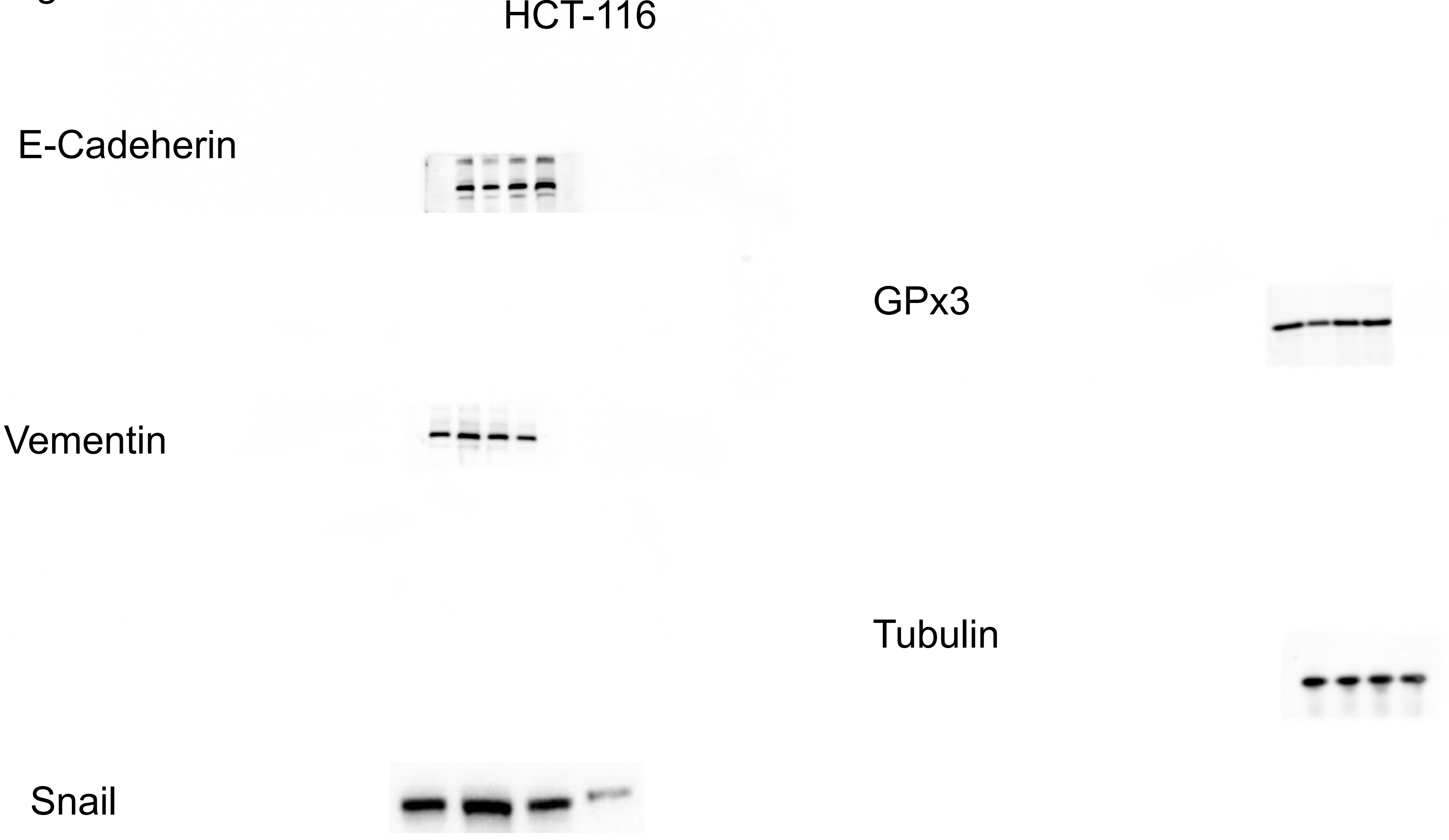

Fig2G

SW480

E-Cadeherin

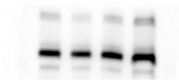

GPx3

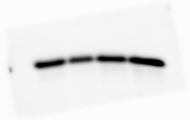

Vementin

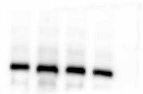

Tubulin

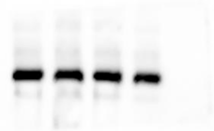

Snail

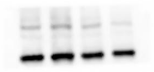

Fig2G

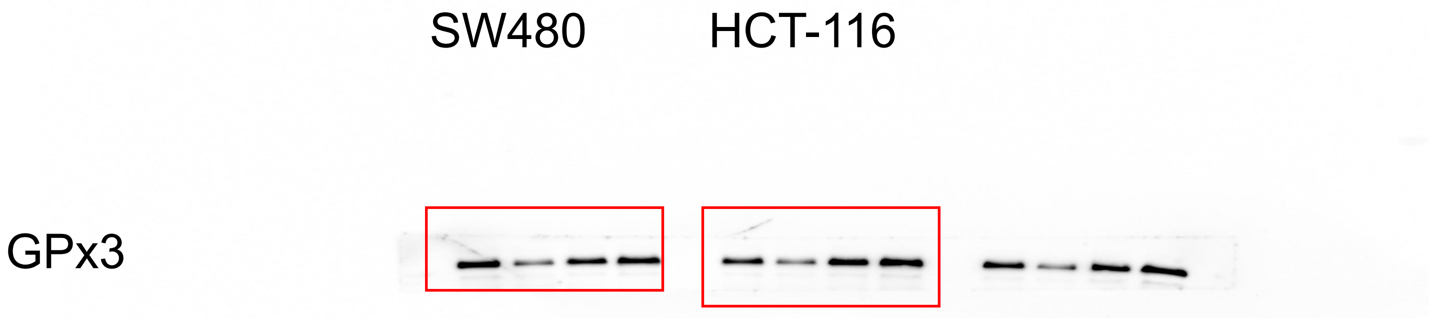

Fig3补充

HCT-116

SW480

LC3

β-actin

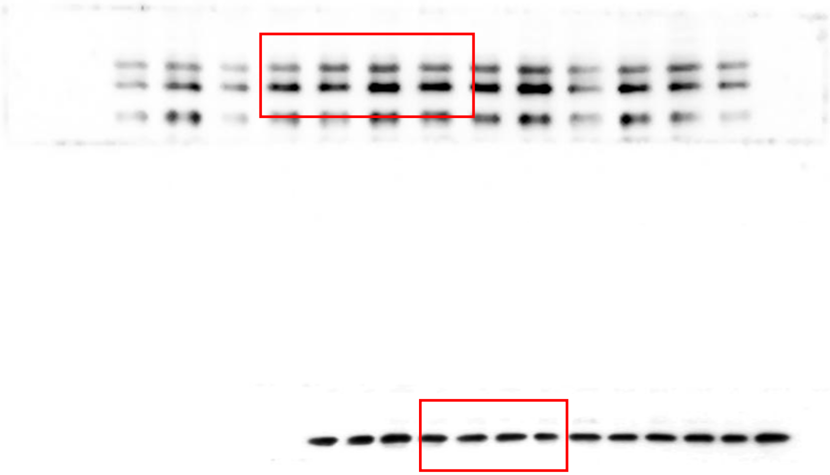

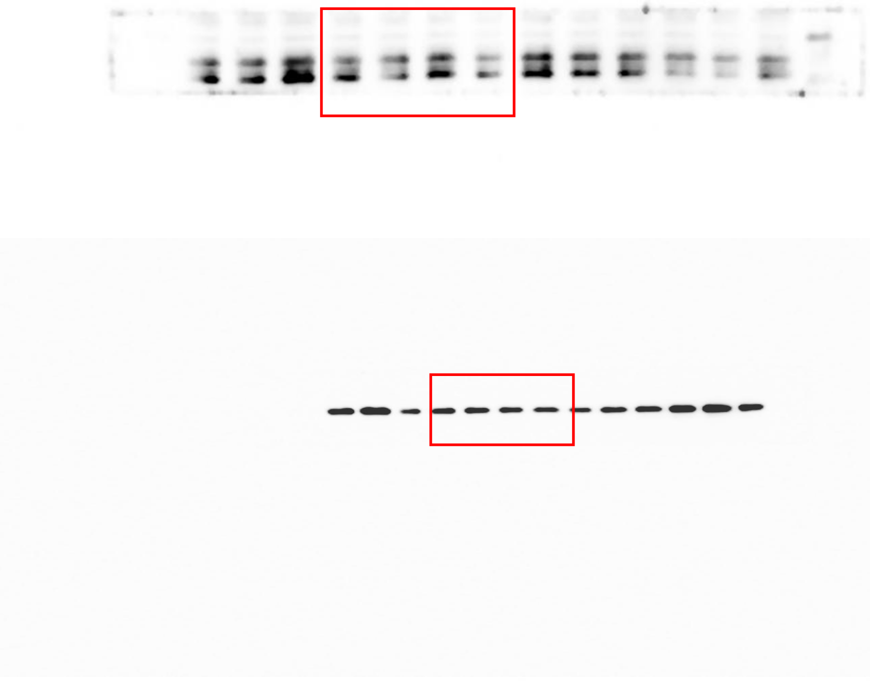

FIG4E

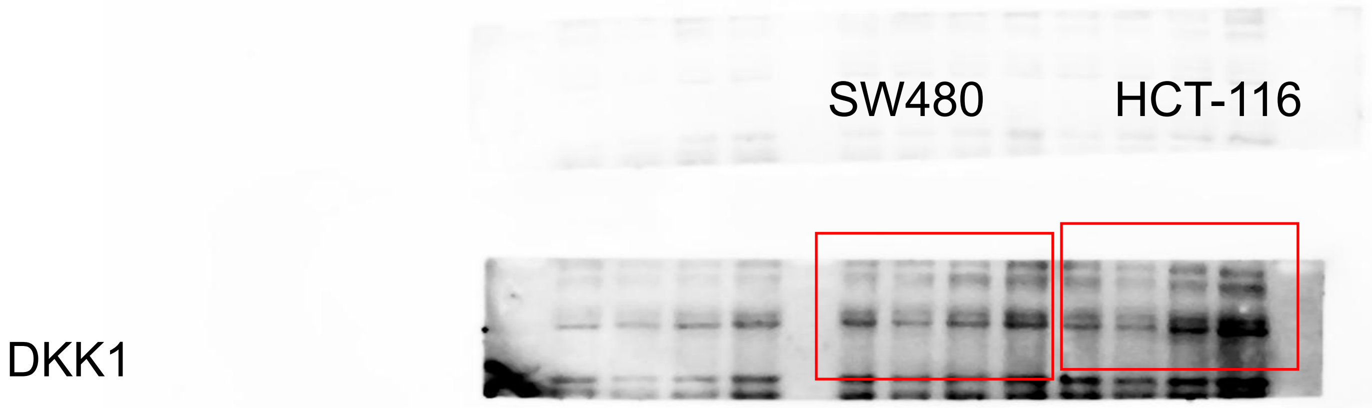

FIG4E

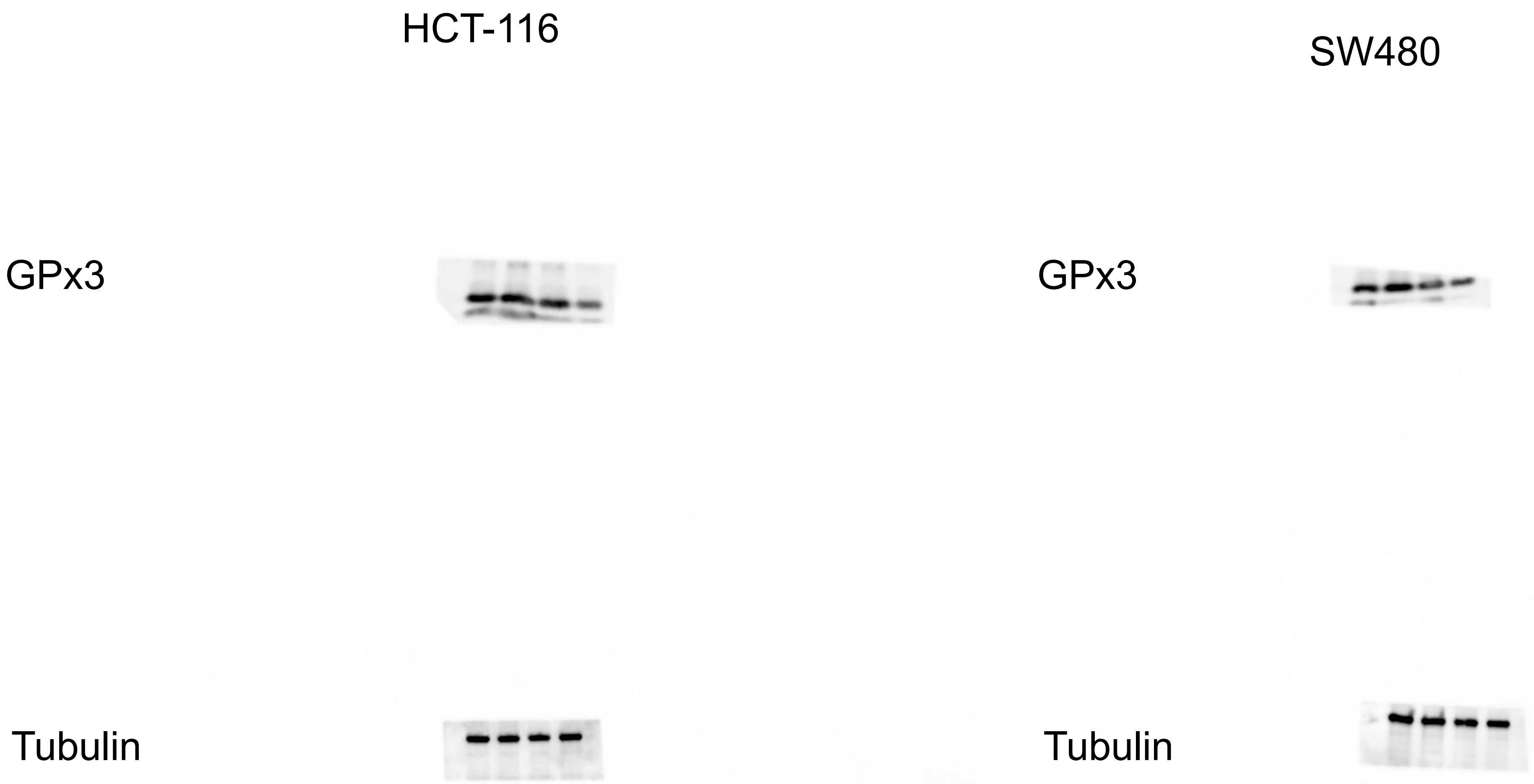

FIG4I

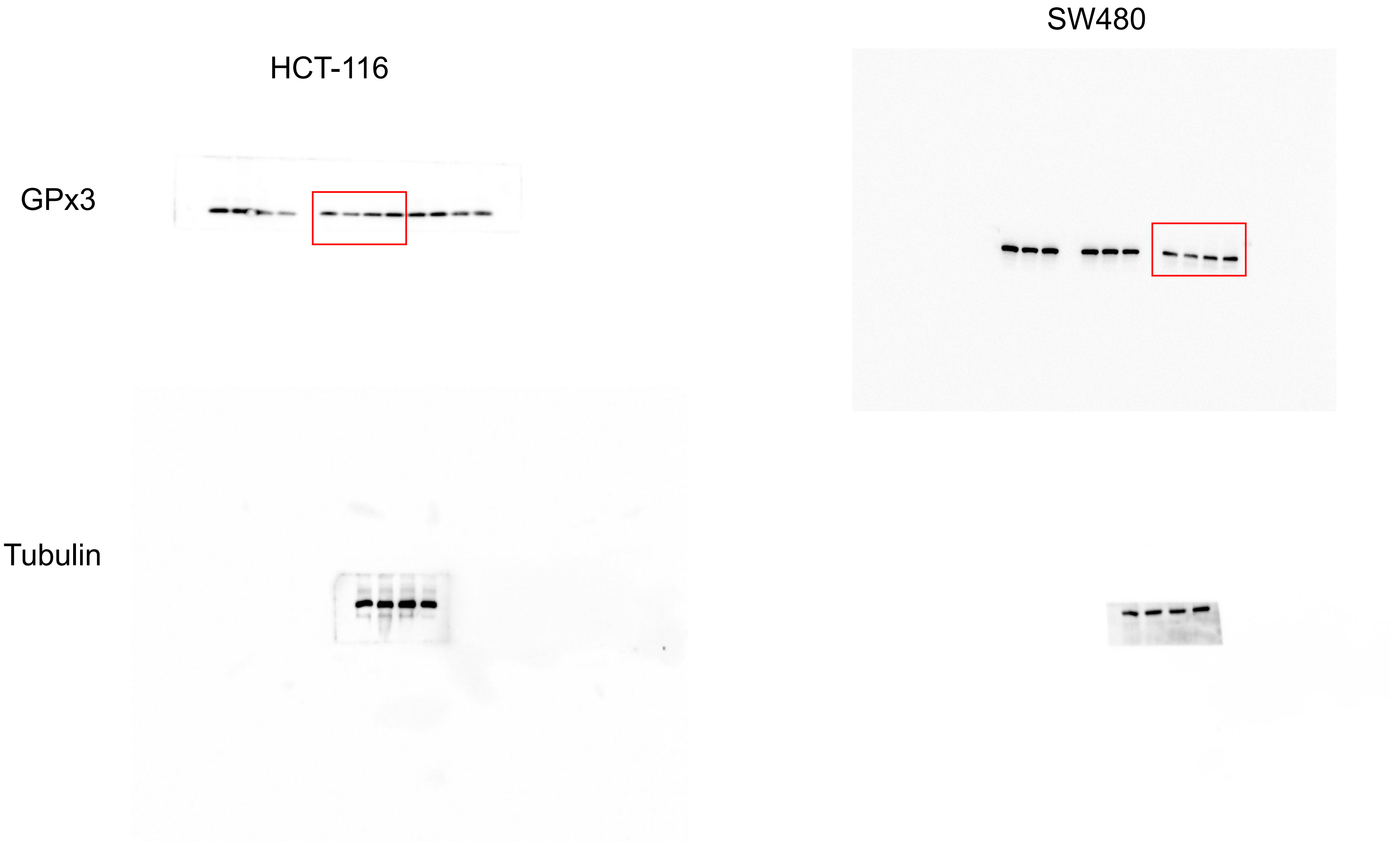

FIG4I

SW480

β-catenin

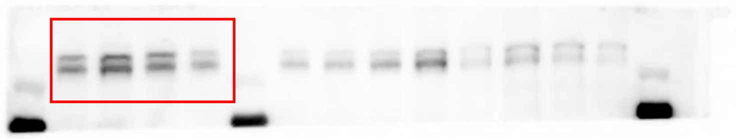

HCT-116

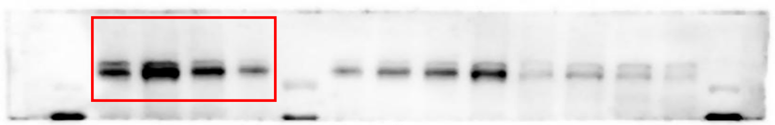

FIG5D

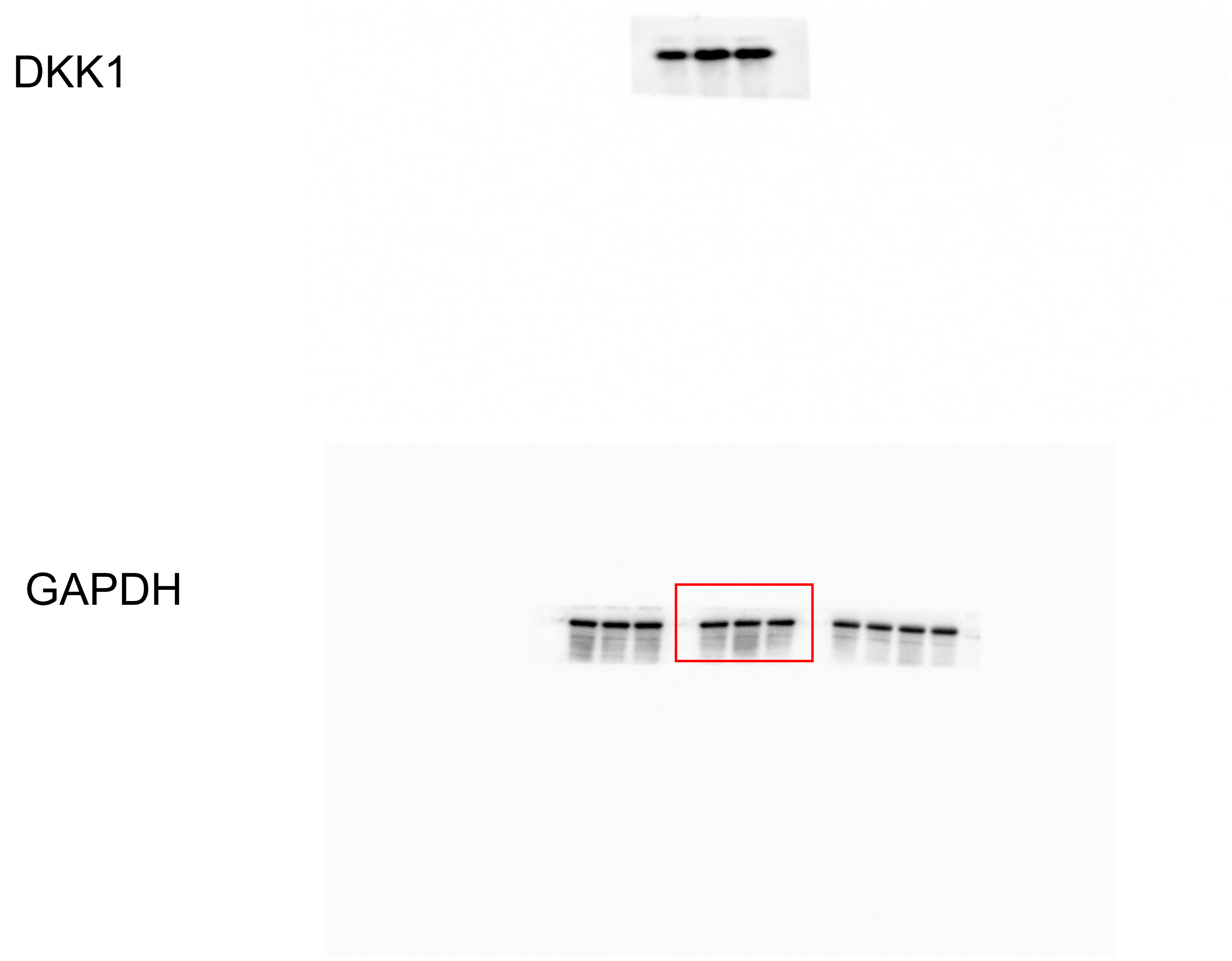

FIG5F

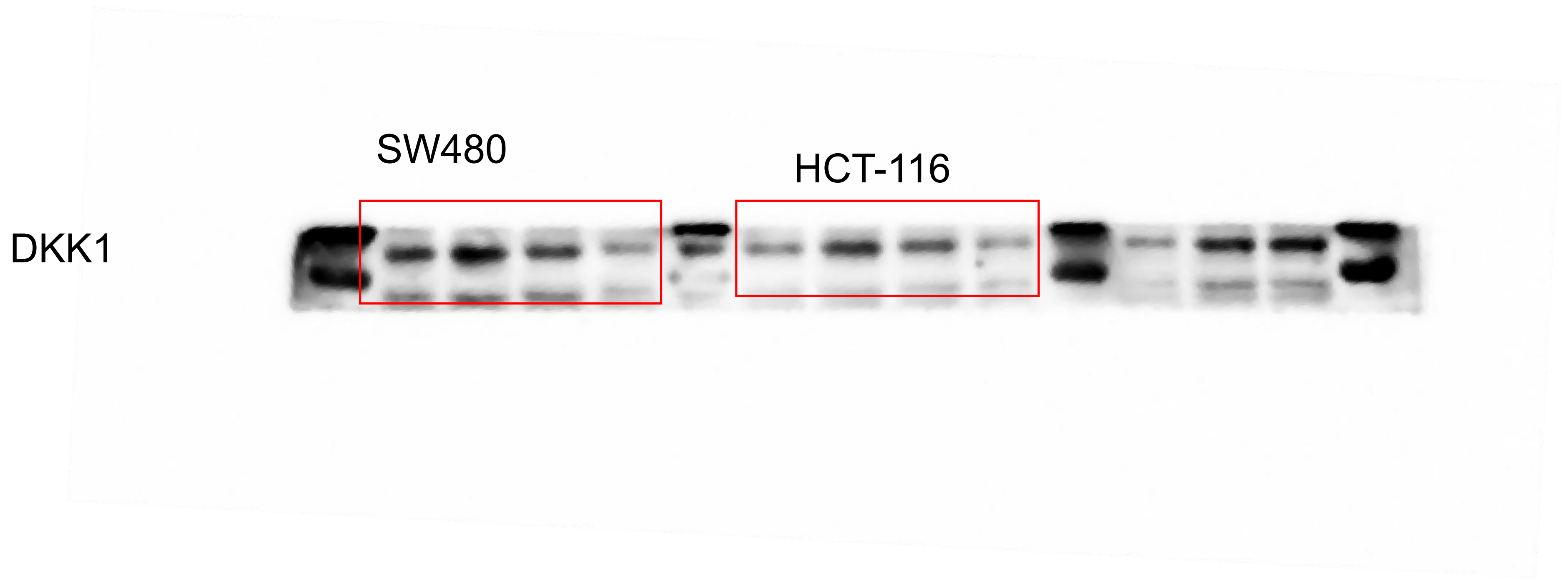

FIG5F

HCT-116

SW480

Tubulin

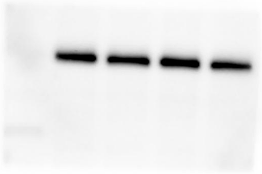

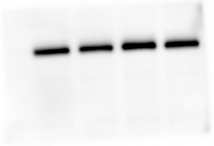

FIG5J

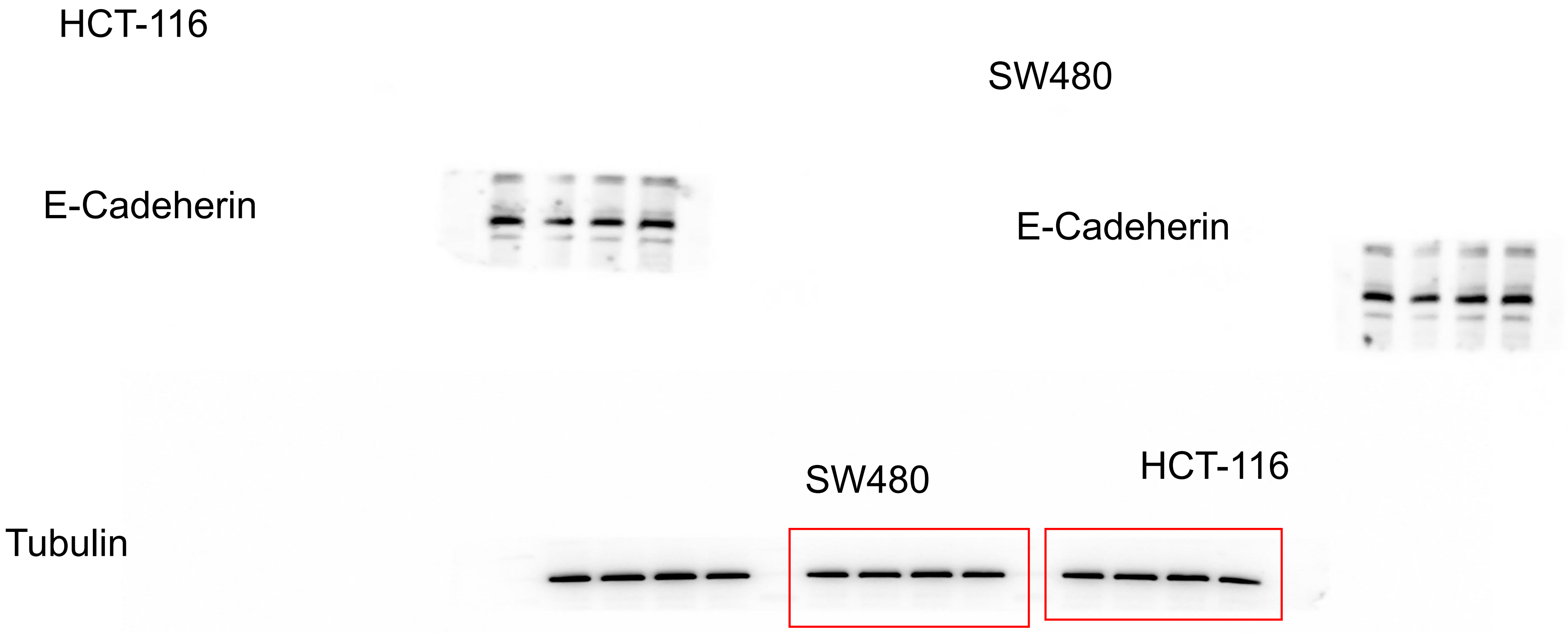

FIG5J

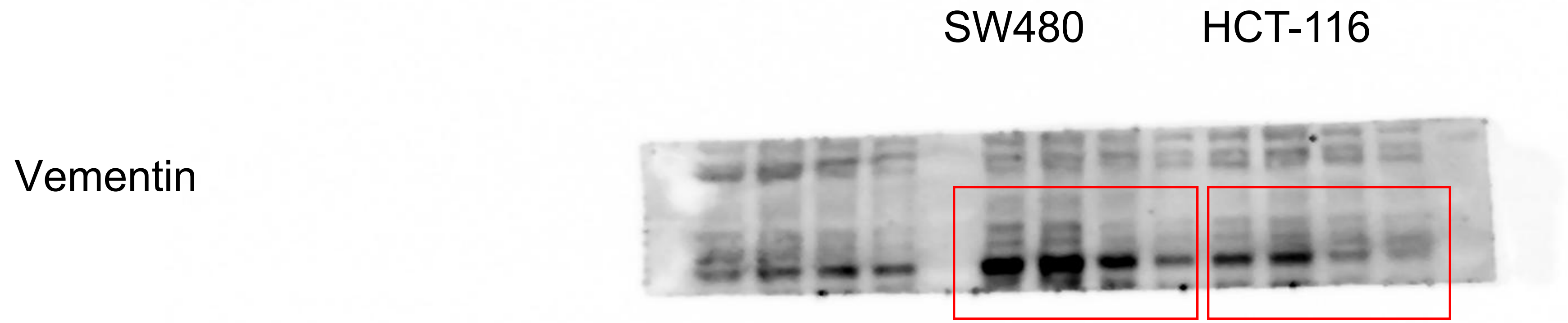

FIG5J

DKK1

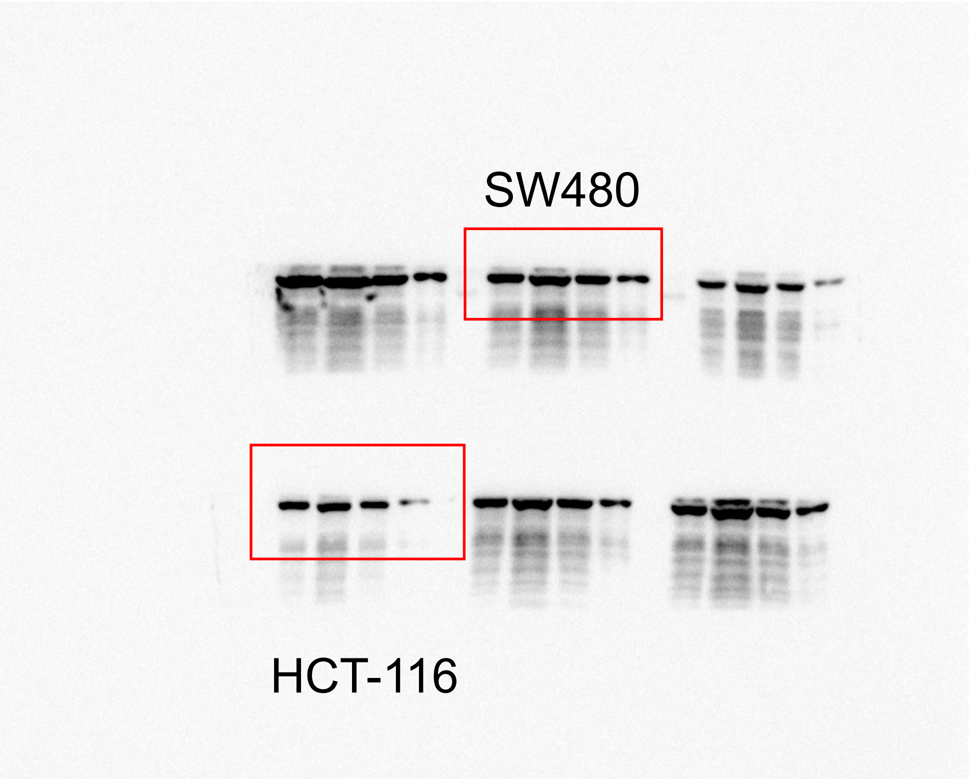

FIG5J

SW480 HCT-116

Snail

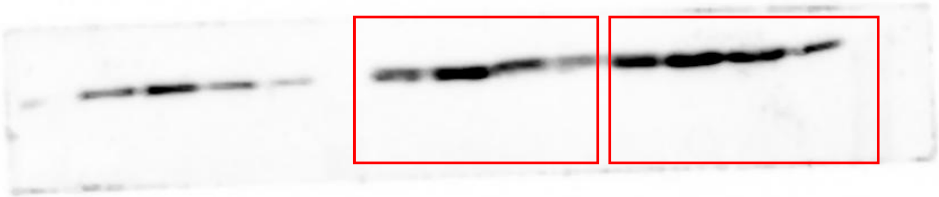

FIG6I

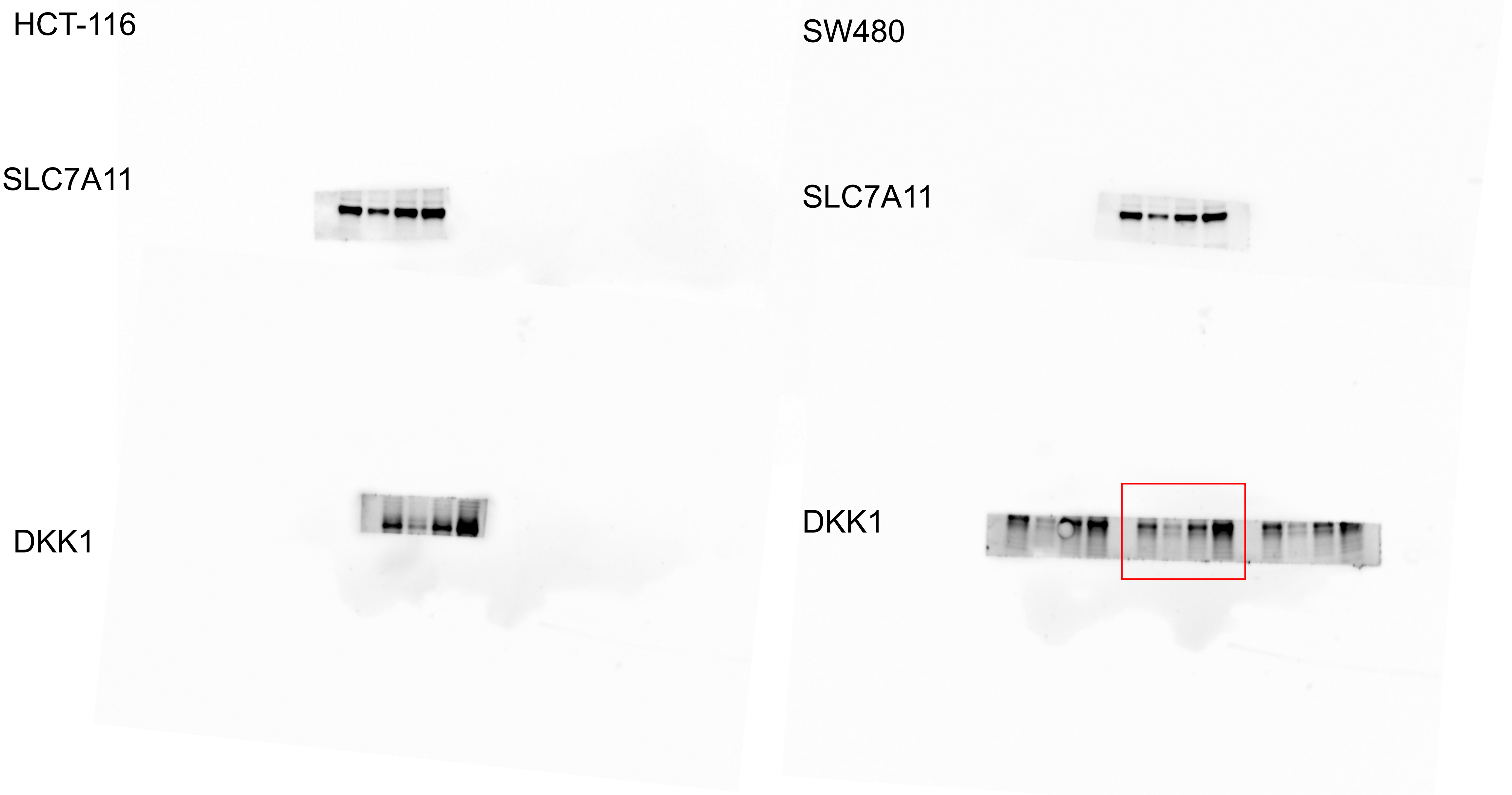

FIG6J

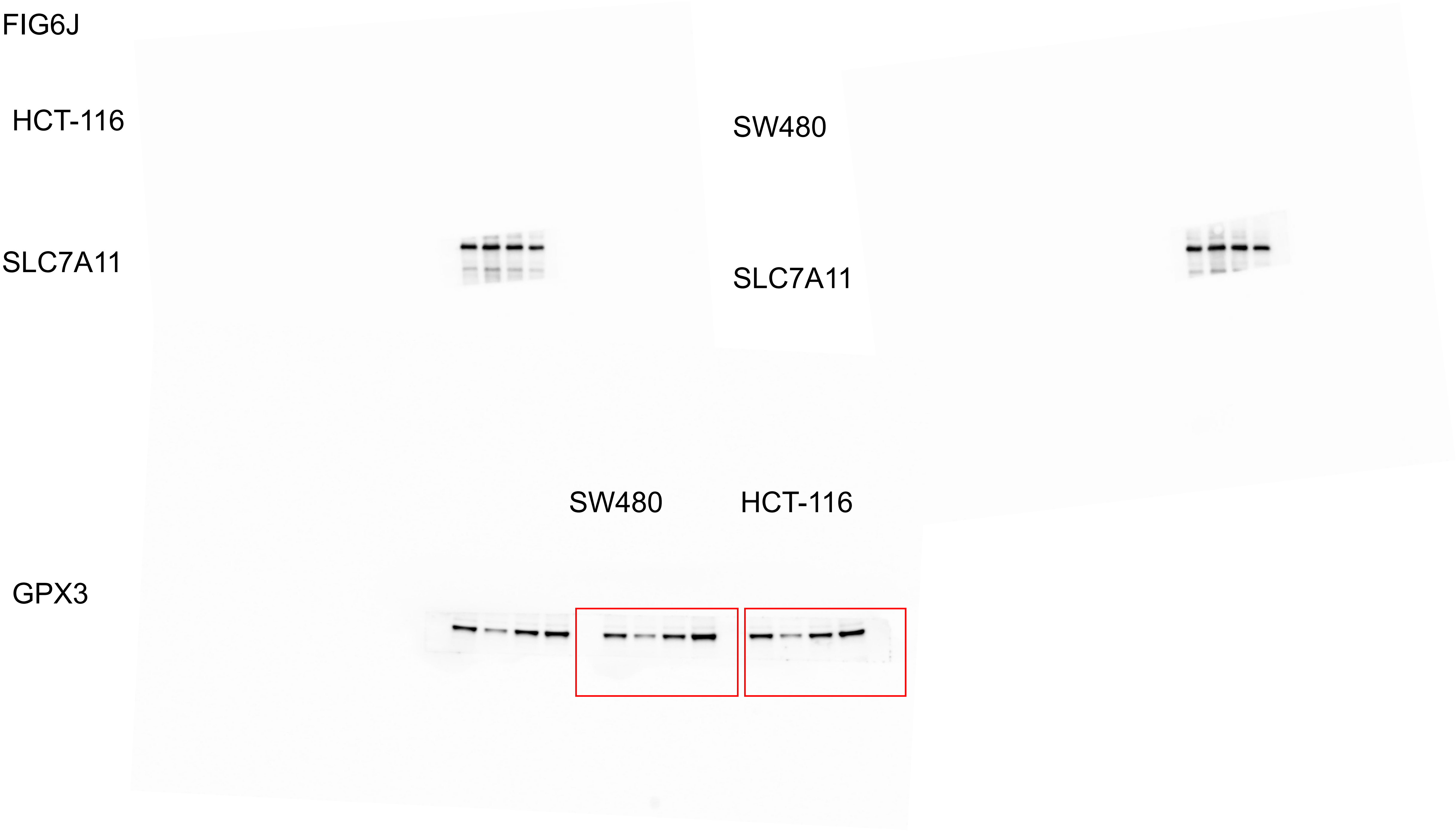

FIG6J

Tubulin

SW480

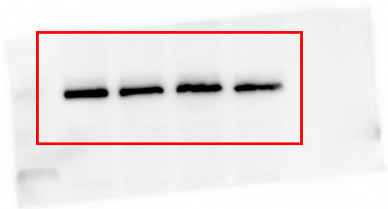

HCT-116

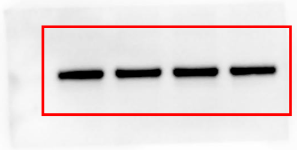

FIG7F

DKK1

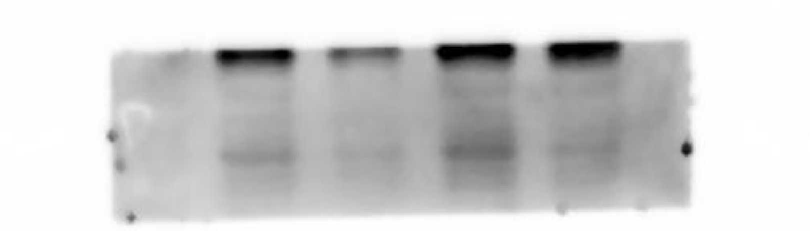

GPx3

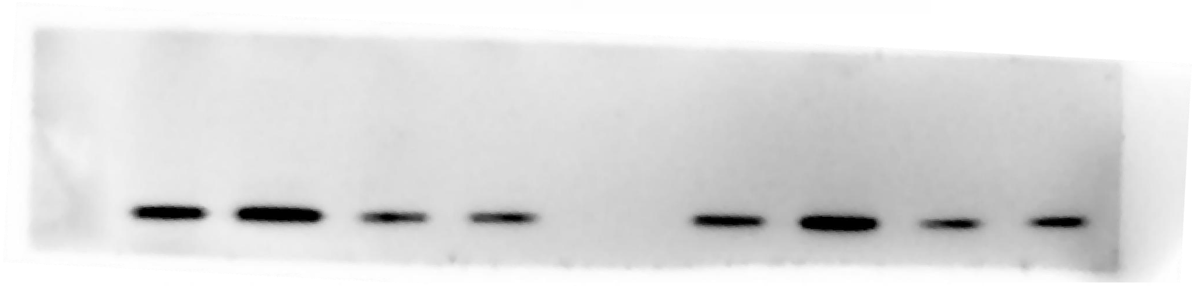

Tubulin

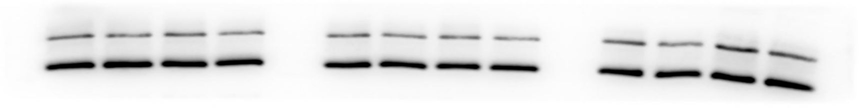

FIG8G

METTL3

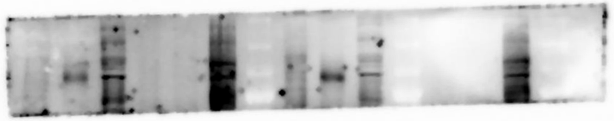

METTL14

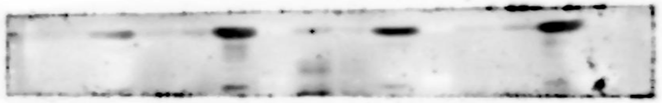

β-actin

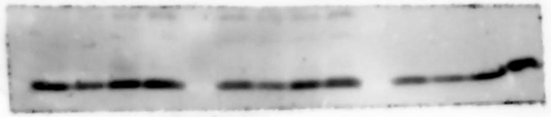

FIG8补METTL14

METTL14

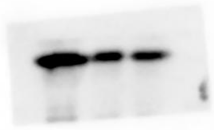

GAPDH

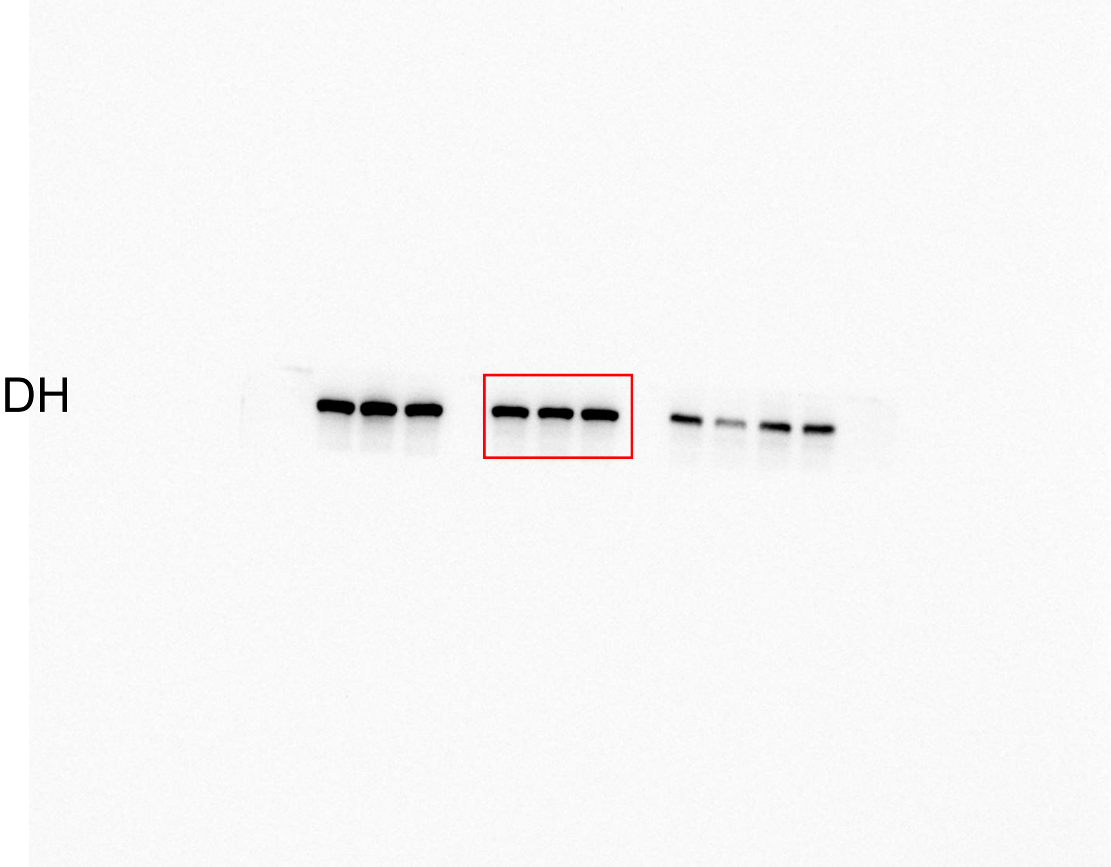

Supplement: Supplementary 1 — Figs. S1 to S7 Uncropped Western Blot Plasmid Information List of Abbreviations [file research.1273.f1.zip › Uncropped_Western_Blot.pdf]
