# Supplementary figures and images for: Methyltransferase-like 3/14-mediated m6A Silencing of GPx3 Drives Lipophagy Dysfunction and Ferroptosis Resistance in Colorectal Cancer
Source: Research (Wash D C). 2026 May 11;9:1273. doi: 10.34133/research.1273 (PMC13158459; doi:10.34133/research.1273)

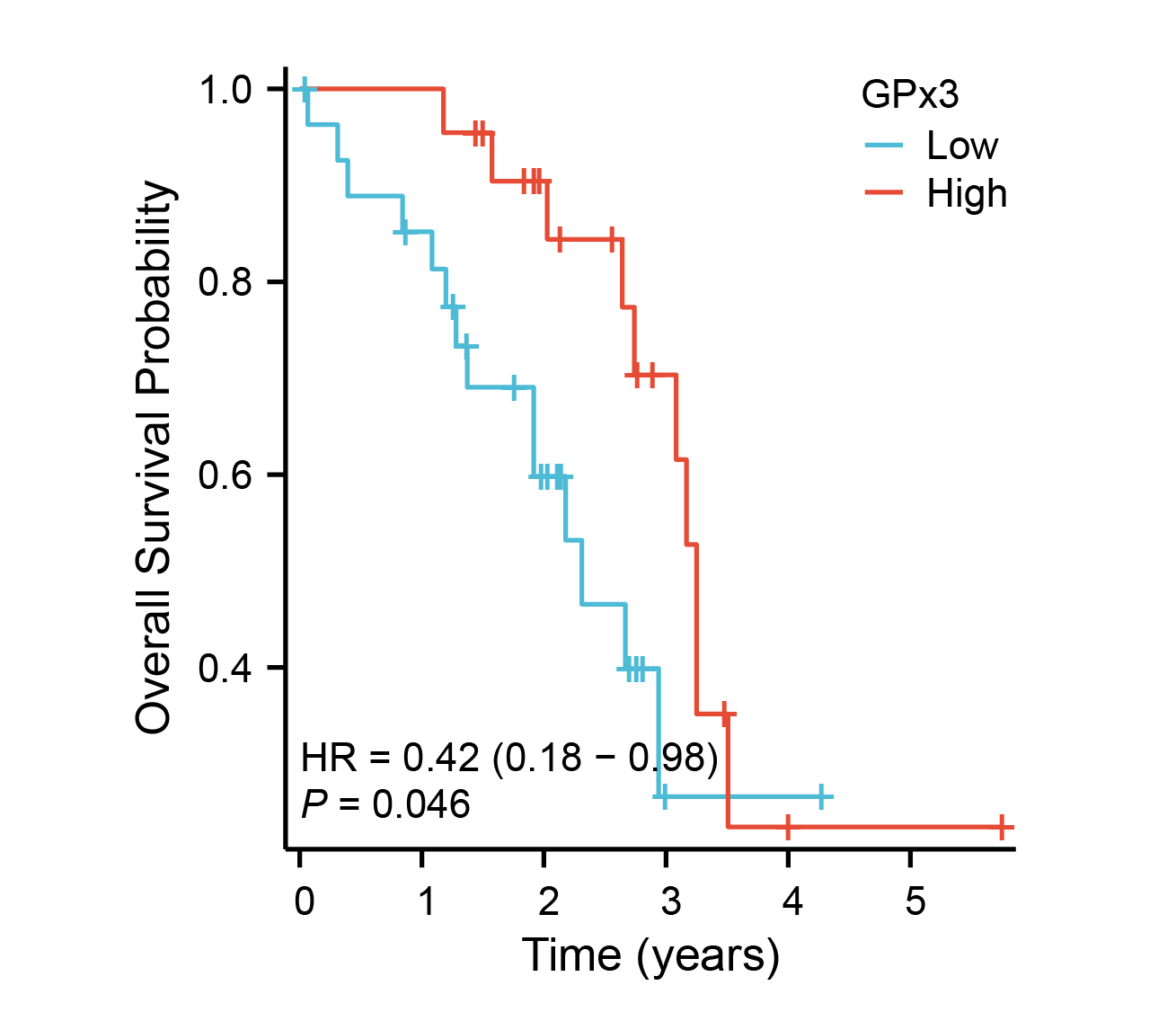

Supplement: Supplementary 1 — Figs. S1 to S7 Uncropped Western Blot Plasmid Information List of Abbreviations [file research.1273.f1.zip › Supplementary Fig._1.tif]

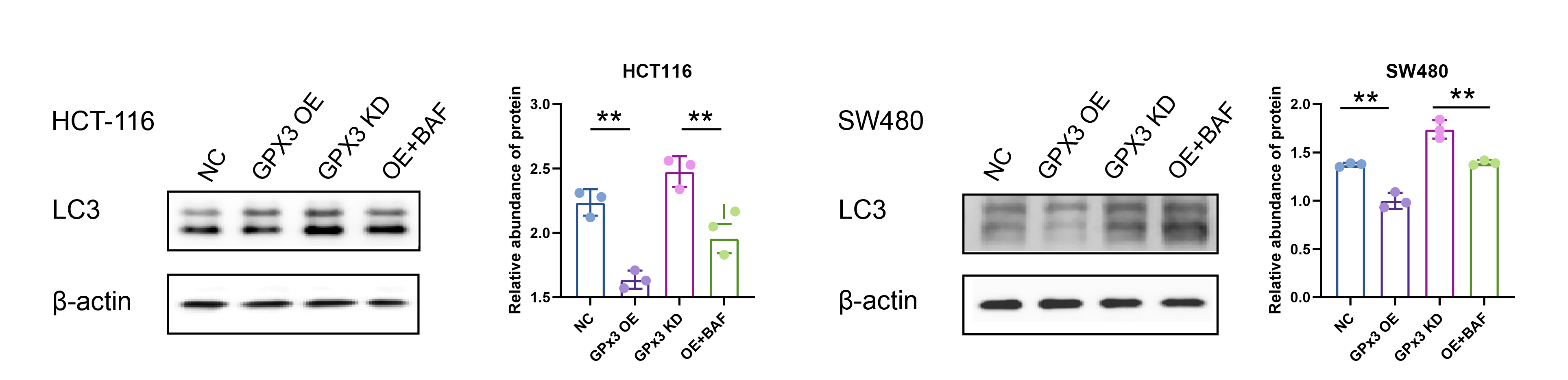

Supplement: Supplementary 1 — Figs. S1 to S7 Uncropped Western Blot Plasmid Information List of Abbreviations [file research.1273.f1.zip › Supplementary Fig._2.tif]

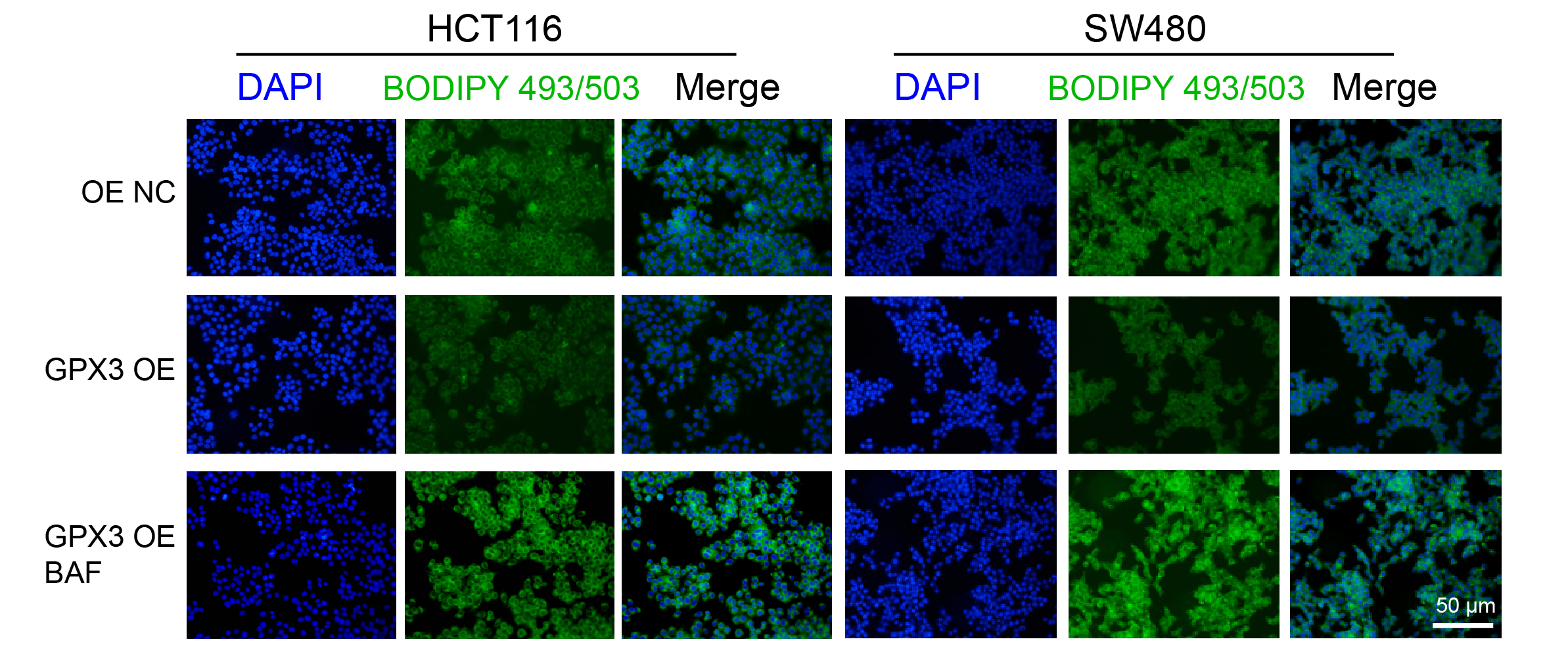

Supplement: Supplementary 1 — Figs. S1 to S7 Uncropped Western Blot Plasmid Information List of Abbreviations [file research.1273.f1.zip › Supplementary Fig._3.tif]

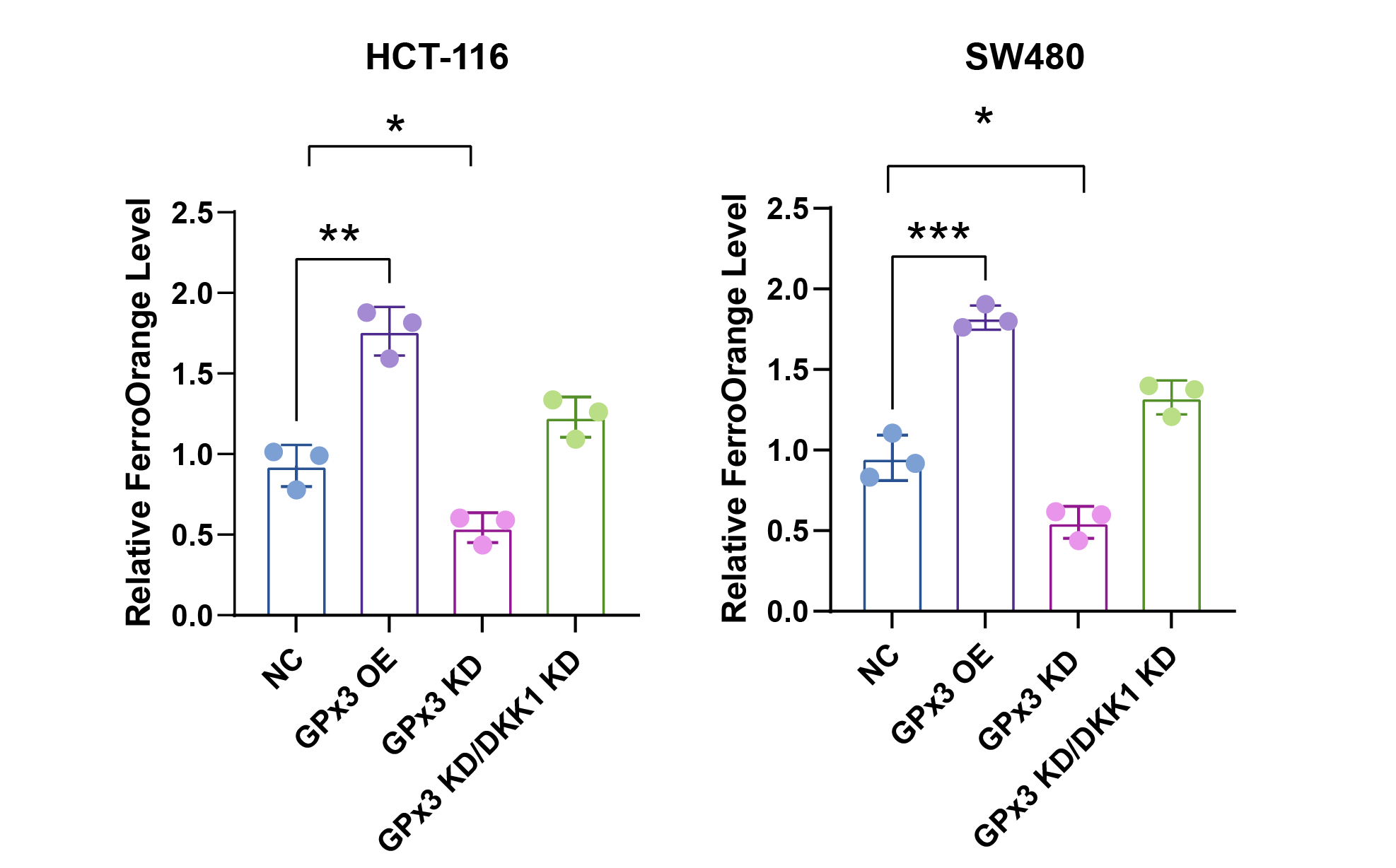

Supplement: Supplementary 1 — Figs. S1 to S7 Uncropped Western Blot Plasmid Information List of Abbreviations [file research.1273.f1.zip › Supplementary Fig._4.tif]

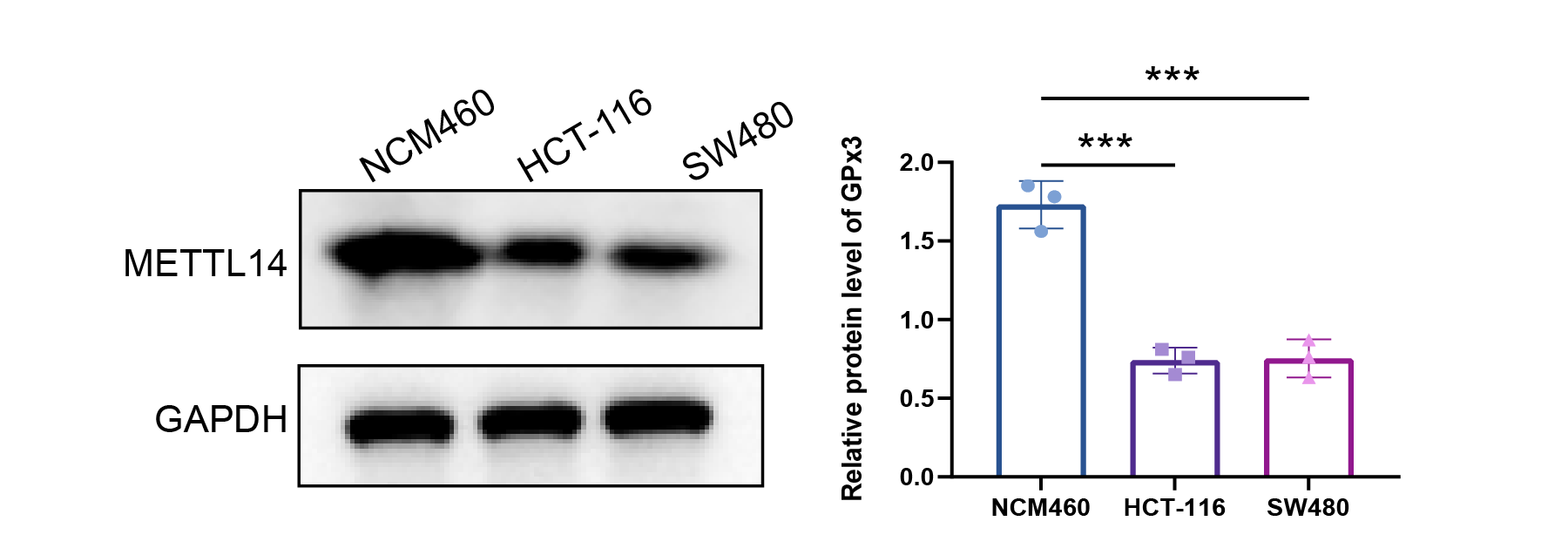

Supplement: Supplementary 1 — Figs. S1 to S7 Uncropped Western Blot Plasmid Information List of Abbreviations [file research.1273.f1.zip › Supplementary Fig._5.tif]

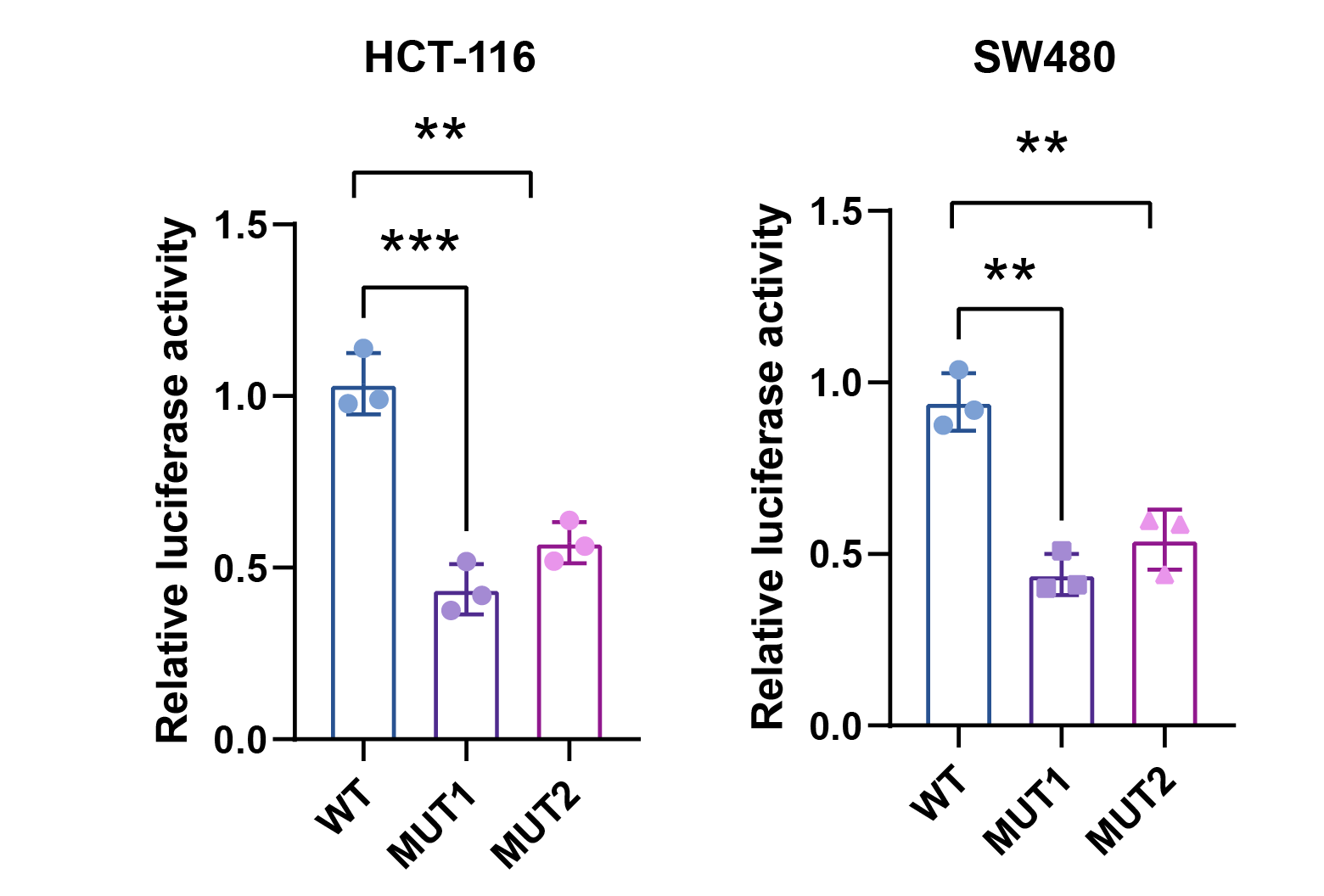

Supplement: Supplementary 1 — Figs. S1 to S7 Uncropped Western Blot Plasmid Information List of Abbreviations [file research.1273.f1.zip › Supplementary Fig._6.tif]

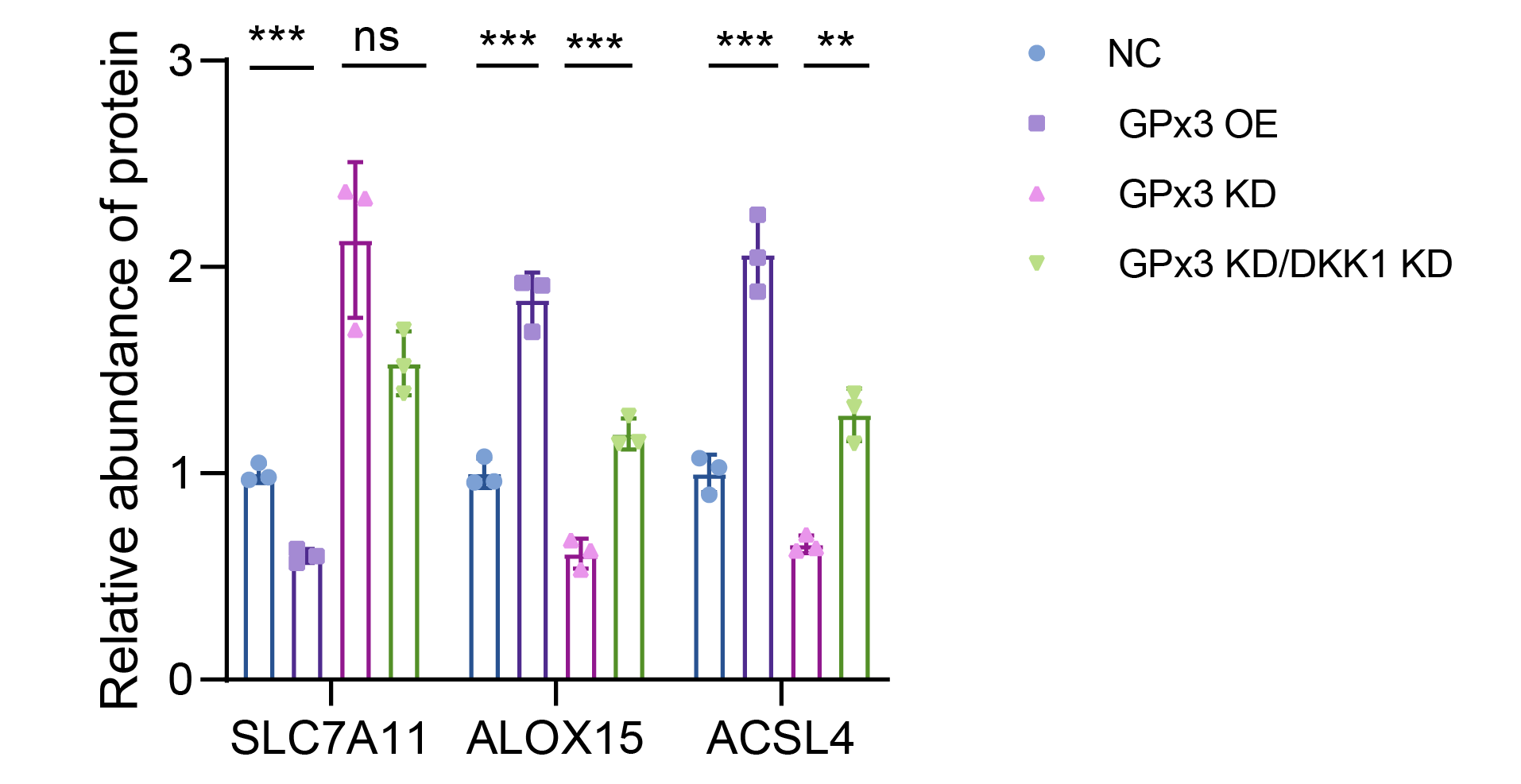

Supplement: Supplementary 1 — Figs. S1 to S7 Uncropped Western Blot Plasmid Information List of Abbreviations [file research.1273.f1.zip › Supplementary Fig._7.tif]
